# Novel Synthesis of the Antifungal Cyclic Lipopeptide Iturin A and Its Fluorinated Analog for Structure‐Activity Relationship Studies

**DOI:** 10.1002/chem.202501341

**Published:** 2025-07-25

**Authors:** Periklis Karamanis, Matthew Kiernan, Jimmy Muldoon, Finn Doyle, Paul Evans, Cormac D. Murphy, Marina Rubini

**Affiliations:** ^1^ School of Chemistry University College Dublin Dublin Ireland; ^2^ BiOrbic Bioeconomy SFI Research Centre University College Dublin Dublin Ireland; ^3^ School of Biomolecular and Biomedical Science University College Dublin Dublin Ireland; ^4^ Conway Institute for Biomolecular and Biomedical Research University College Dublin Dublin Ireland

**Keywords:** antifungal agents, cyclic lipopeptides, fluorinated peptides, fluorine NMR, iturin A, peptide cyclization, peptides

## Abstract

The rapid development of antifungal resistance poses a threat to the health and agricultural sectors. Iturin A, a cyclic lipopeptide with pronounced antifungal properties produced by *Bacillus sp*., holds promise against several pathogens. Here, a novel synthesis of iturin A is presented, which enables access to different analogs for the study of its mode of action. The route includes the enantioselective synthesis of the β‐amino fatty acid present in the lipopeptide structure, followed by solid‐phase peptide synthesis and on‐resin cyclization. This robust synthesis was used to obtain natural iturin A. Furthermore, the synthesis of two analogs is described: an epimer with an inverted stereochemistry of the β‐amino fatty acid, which was designed to shed light on the role of this stereocenter on iturin A's bioactivity, and a monofluorinated analog to assess fluorination's impact on bioactivity and as a fluorine NMR probe for mechanistic studies. Antifungal assays against *Candida albicans* and *Fusarium graminearum* showed that the epimer of iturin A lost all bioactivity, while the monofluorinated analog retained the bioactivity of the natural compound, thus confirming its potential as an NMR probe.

## Graphical abstract

Iturin A is an antifungal lipopeptide with a wide range of applications. In this study, we present a novel synthesis of iturin A via enantiomeric synthesis of the β‐amino fatty acid and on‐resin cyclization. We also obtained the (*S*)‐epimer of iturin A, showing a dramatic loss of bioactivity, and a monofluorinated analog, which retained the bioactivity of the natural compound and can be a useful fluorine NMR probe for mechanistic studies.

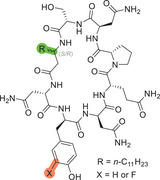

## 1 Introduction

The threat of fungal infections and the rise of antifungal resistance pose a continuous hazard to human health, food safety, and crop yield.^[^ ^1^, ^2^, ^3^ ^]^ Soil bacteria, such as *Bacillus* sp., are used as biocontrol agents, for example Serenade, and have been regarded as a sustainable replacement for chemical pesticides.^[^ ^4^ ^]^ Efforts to identify the specific compounds from *B. subtilis* that display antimicrobial activity have led to the discovery of a wide array of non‐ribosomal cyclic lipopeptides. These belong to the iturin, fengycin, and surfactin families.^[^ ^4^, ^5^ ^]^ A number of structurally related compounds belonging to the broader iturin family have also been identified (iturins, bacillomycins, mycosubtylin, and mojavensin).^[^ ^6^ ^]^ One of these compounds, iturin A, displays particularly potent antifungal activity against several fungal pathogens^[^ ^7^, ^8^, ^9^ ^]^ along with potential antiviral^[^ ^10^ ^]^ antibacterial,^[^ ^11^ ^]^ and anticancer^[^ ^12^ ^]^ properties. Therefore, it has been proposed that, owing to its broad biological activity, iturin A may find applications in different fields, including the health, food, and agricultural sectors.^[^ ^13^ ^]^

**Figure 1:**
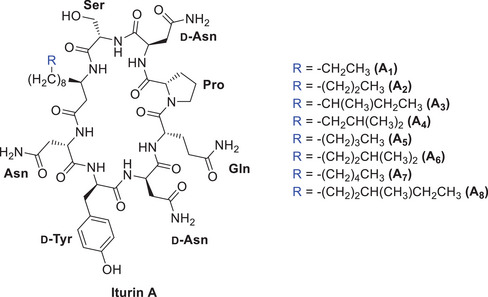
Structure of iturin A isoforms, produced by *Bacillus subtilis*.

Iturin A consists of seven α‐amino acids with a conserved stereochemical pattern in its sequence and a β‐amino fatty acid, also known as iturinic acid, with varying alkyl chain length (C_14_‐C_17_) and (*R*) configuration at the β‐amino fatty acid stereogenic center.^[^ ^14^, ^15^ ^]^ The alkyl side‐chain can be linear or can be branched toward its terminus (iso or anteiso), and in total, eight isoforms (A_1_‐A_8_) are recognised (Figure 1).^[^ ^13^ ^]^

The mechanism of action of iturin A is thought to involve hydrogen bonding between the hydroxy group of the D‐tyrosine residue and the sterols present in the fungal membrane. This leads to the penetration by the hydrophobic tail into the cytoplasmic membrane, causing pore formation and cell leakage.^[^ ^16^, ^17^ ^]^ In fact, it has been reported that *O*‐methylation of iturin A's tyrosine residue results in a dramatic decrease in bioactivity.^[^ ^18^ ^]^ Furthermore, the replacement of iturinic acid with β‐alanine showed complete loss of bioactivity, highlighting the importance of the lipophilic alkyl side‐chain.^[^ ^19^ ^]^ Related to this, the D‐Tyr residue is strictly conserved among all of the iturinic lipopeptides, whilst the alkyl side‐chain is present in all of the lipopeptide families produced by *B. subtilis*.^[^ ^6^, ^14^ ^]^ Other mechanistic studies have clearly demonstrated the link between the induction of oxidative stress and accumulation of reactive oxygen species and the ability of iturin A to disrupt fungal growth.^[^ ^20^, ^21^ ^]^

Synergistic effects between iturin A and surfactin, concerning haemolytic and antifungal activity, have also been reported and have been attributed to the formation of mixed micelles.^[^ ^22^, ^23^, ^24^ ^]^

While there have been numerous efforts directed toward the optimization of the bacterial production of iturin A,^[^ ^25^, ^26^, ^27^ ^]^ few studies concerning the total synthesis of iturin A and its analogs have been reported. The only synthesis of iturin A_2_ was described in 1996, and it involved a combination of solid‐phase peptide synthesis techniques with a subsequent cyclization in the liquid phase.^[^ ^19^ ^]^ Later attempts at simplifying the lipopeptide structure by replacing the β‐amino acid with an α‐amino acid, via cysteine lipidation through native chemical ligation techniques, yielded analogs that were inactive under the tested conditions.^[^ ^28^ ^]^ These synthetic analogs displayed an ester moiety in the alkyl side‐chain that might be responsible for the decrease in hydrophobicity of the lipopeptide, thus negatively impacting its bioactivity. Inspired by this study, we previously described the synthesis of iturin A analogs containing an alkylated cysteine. The non‐ester‐based compounds proved to be active against *Fusarium graminearum*, albeit less so than the natural lipopeptide, showing the importance of the β‐amino acid for bioactivity.^[^ ^29^ ^]^

**Figure 2:**
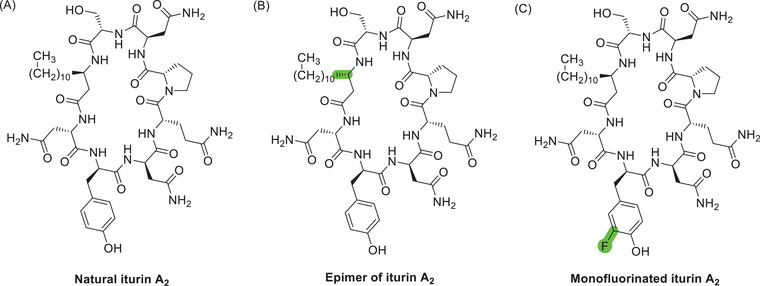
Structures of the lipopeptides synthesised in this work. (A) Natural iturin A_2_, (B) Epimer of iturin A_2_, (C) Monofluorinated iturin A_2_.

Here we report an improved total synthesis of iturin A_2_ (Figure 2A), featuring fluorenylmethyloxycarbonyl (Fmoc) solid‐phase peptide synthesis (SPPS) techniques and an on‐resin cyclization step, with the use of appropriate orthogonal protecting groups.^[^ ^30^ ^]^ Furthermore, we employed this robust synthetic route to investigate both the influence of the stereochemistry of the iturinic acid (Figure 2B) on the lipopeptide's bioactivity and the effect of fluorination at the ortho position of the phenol ring on the hydrogen bond capacity of the crucial phenolic hydroxy group on the D‐Tyr residue. (Figure 2C).^[^ ^16^, ^19^ ^]^ Fluorine could provide enhanced metabolic stability and, importantly, could serve as a ^19^F NMR probe to shed light on the mode of action of the lipopeptide.^[^ ^31^, ^32^, ^33^ ^]^

## 2 Results and Discussion

### 2.1 Synthesis of the Iturinic Acid

**Figure 3:**
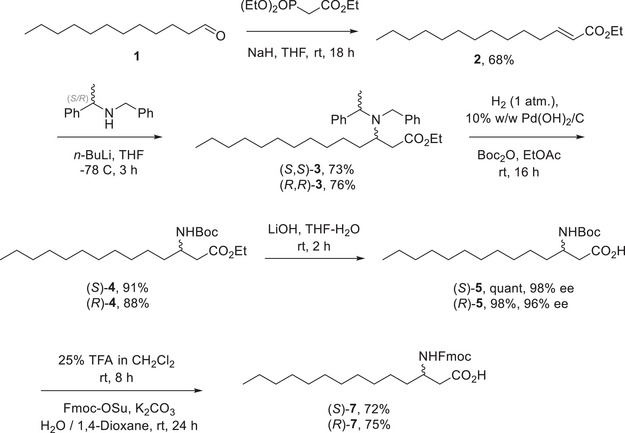
Enantioselective synthesis of Boc and Fmoc protected iturinic acid ((*R*)‐**5**; (*R*)‐**7**) and of its enantiomer ((*S*)‐**5**; (*S*)‐**7**).

To the best of our knowledge, only one synthetic route for the total synthesis of natural iturin A is reported in the literature.^[^ ^19^ ^]^ In part, this is due to the limited and laborious methods available to synthesise the required β‐amino fatty acid (iturinic acid).^[^ ^34^, ^35^, ^36^ ^]^ Homochiral lithium amides, developed by Davies et. al., have been used for the diastereoselective synthesis of β‐amino acids with good yields and excellent enantioselectivity. Therefore, they were deemed an ideal chiral auxiliary‐based approach for the synthesis of iturinic acid and of its enantiomer (Figure 3).^[^ ^37^, ^38^ ^]^

The first step included the synthesis of the α,β‐unsaturated ester **2**, through a Horner—Wadsworth–Emmons reaction.^[^ ^39^ ^]^ The enantiomeric lithium amides were then introduced, giving the two separate diastereomers (*S*,*S*)‐**3** and (*R*,*R*)‐**3** in good yields.^[^ ^38^ ^]^ Dibenzyl removal via hydrogenation, with in situ *tert*‐butyloxycarbonyl (Boc) protection of the amine, followed by saponification, gave the final products (*S*)‐**5** and (*R*)‐**5** in excellent yields. The determination of the enantiomeric excess of the final products was performed via normal phase chiral HPLC analysis, after benzylation of the carboxylic acids ((*R*)‐**6** and (*S*)‐**6**, see Supporting Information for details), and was determined to be excellent.

The β‐amino fatty acids were initially synthesised with a Boc protecting group to allow for the simultaneous global deprotection of the peptide and cleavage from the resin, with a subsequent head‐to‐tail cyclization in liquid phase under high dilution.^[^ ^29^ ^]^ Fmoc protected β‐amino fatty acids ((*R*)‐**7** and (*S*)‐**7**) were also obtained, using previously reported methods.^[^ ^40^ ^]^ The Fmoc protection was performed to open up alternative synthetic routes, such as an on‐resin cyclization reaction (described below).^[^ ^41^ ^]^ In relation to the formation of (*S*)‐**7** and (*R*)‐**7**, the presence of a small amount of Fmoc‐β‐Ala‐OH was observed, via LC‐MS, in the crude mixture of the reactions. As reported, this is likely due to the use of the Fmoc‐OSu reagent.^[^ ^42^ ^]^

### 2.2 Synthesis of Iturin A_2_ and of Its Analogs

We previously reported the synthesis of *S*‐alkyl iturin A analogs via Fmoc‐SPPS, followed by a head‐to‐tail cyclization of the unprotected linear peptide, performed under high dilution in liquid phase.^[^ ^29^ ^]^ This methodology is suitable for the synthesis of iturin A, possibly due to its adequate size (smaller peptides have been reported to be harder to cyclise) and pre‐organization of the peptide backbone.^[^ ^43^ ^]^ Following this strategy, natural iturin A and its non‐natural epimer (Figure 2A,B) were obtained in satisfactory overall yields (8–10%). However, this approach could not be reliably used for the synthesis of the monofluorinated analog (Figure 2C), as a dramatic decrease in overall yield (<2%) was observed. We speculated that this was due to the presence of fluorine on the D‐Tyr residue and that the deprotonated phenol participated in undesirable side reactions, such as macrolactonization.

LC‐MS analysis of the main side‐product showed an m/z ratio consistent with a guanidinylated macrolactone. N‐terminus guanidinylation of peptides is a well reported side‐reaction during Fmoc‐SPPS when using uronium/aminium‐based coupling reagents (such as TBTU).^[^ ^44^ ^]^ Furthermore, these coupling reagents have been used for the synthesis of esters from the reaction of phenols and carboxylic acids.^[^ ^45^ ^]^ It is therefore assumed that the formation of the macrolactone between the C‐terminus and the fluorinated phenol favored the guanidinylation of the free amine at the N‐terminus.

**Figure 4:**
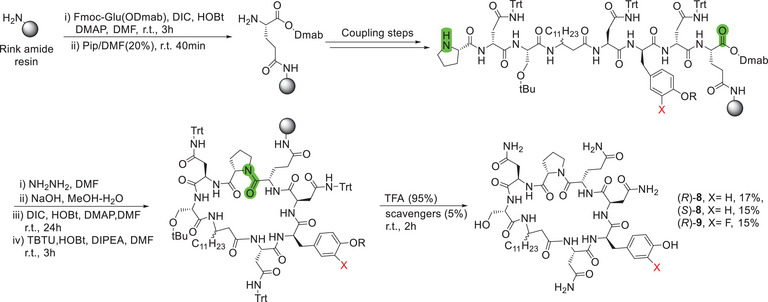
Solid‐phase peptide synthesis and on‐resin cyclization of iturin A analogs. (*R*)‐**8**: natural iturin A_2_, (*S*)‐**8**: epimer of iturin A_2_, (*R*)‐**9**: monofluorinated analog of iturin A_2_. For (*R*)‐**8** and (*S*)‐**8**, R = tBu. For (*R*)‐**9**, R = H.

To overcome this issue, an on‐resin cyclization approach was developed (Figure 4). This approach took advantage of the presence of a glutamine residue in the lipopeptide structure, which allowed the anchoring of the resin on the amino acid side chain. Gratifyingly, higher yields and simplified peptide purification processes were achieved using this route, and it should be mentioned that this strategy has been previously reported for the successful synthesis of fengycin analogs.^[^ ^41^ ^]^ The C‐terminus carboxylic acid was orthogonally protected with Dmab, as it is stable under basic conditions and can be selectively deprotected in a 5% hydrazine solution.^[^ ^46^ ^]^ These mild deprotection conditions made Dmab a suitable choice, as other orthogonal protecting groups (such as allyl esters) have been reported to be more difficult to deprotect.^[^ ^41^ ^]^

**Figure 5:**
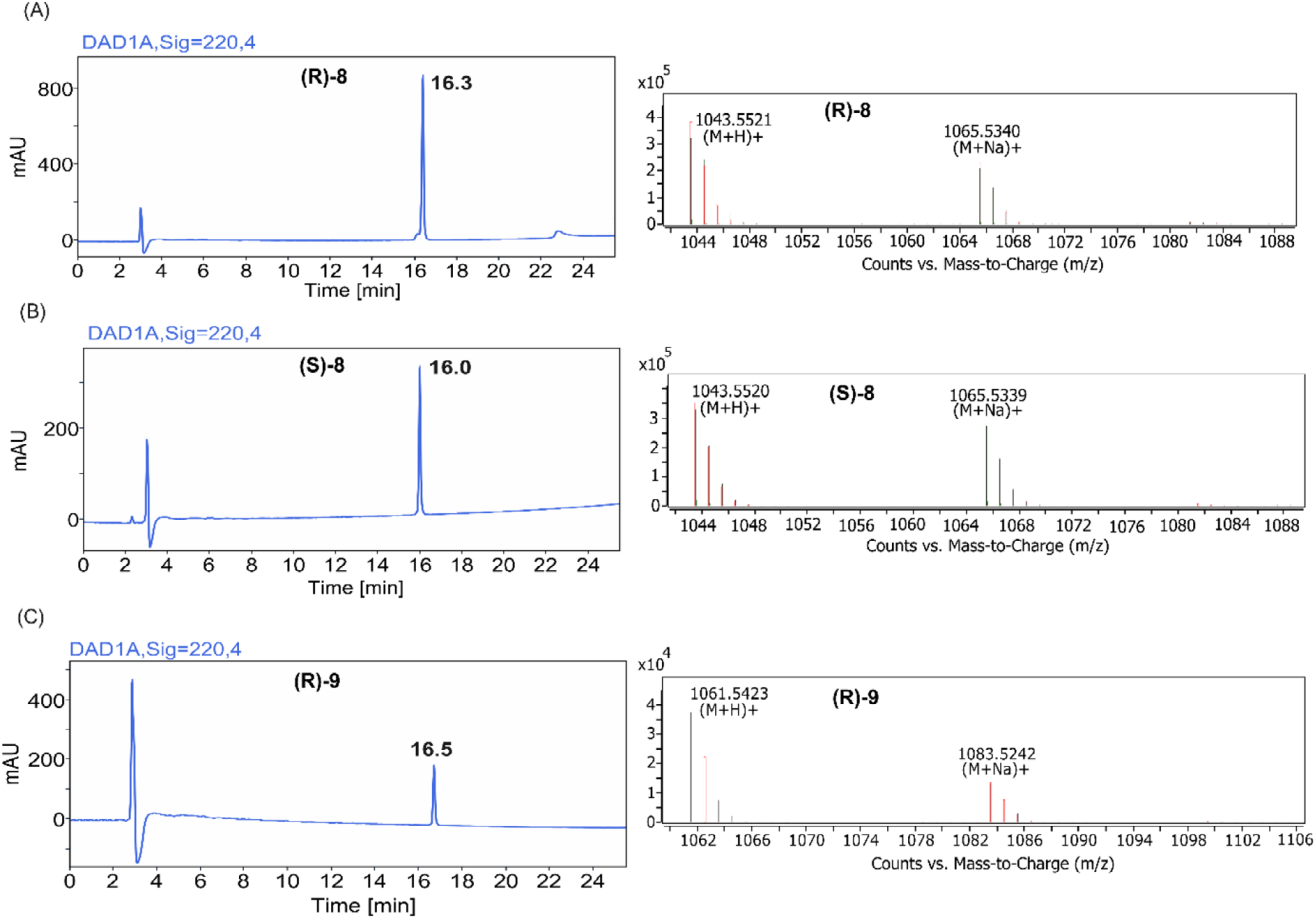
HPLC traces (left) and MS spectra (right) of the purified lipopeptides. (A) (*R*)‐**8**, natural iturin A_2_, (B) (*S*)‐**8**, epimer of iturin A_2_, (C) (*R*)‐**9**, monofluorinated analog of iturin A_2_.

This method afforded the three lipopeptides [(*R*)‐**8**; (*S*)‐**8**; (*R*)‐**9**] in good overall yields and with >95% purity (Figure 5). The desired lipopeptides were the major product observed via LC‐MS analysis. The lack of side‐chain protection on the commercially available monofluorinated tyrosine residue did not decrease the yield or purity of the final product. Interestingly, even though the cyclization occurred on the secondary amine of the proline residue, only traces of the linear peptide precursors were found in the crude mixtures. These results prove the superiority of the on‐resin approach when compared to previously reported liquid‐phase cyclization methods,^[^ ^19^, ^29^ ^]^ especially when introducing potentially reactive, non‐natural amino acids to the structure.

The retention times on the HPLC chromatograms can be indicative of the lipophilicity of the obtained lipopeptides.^[^ ^47^ ^]^ While the variations are quite small, it can be deduced that the addition of fluorine slightly increases the lipophilicity of iturin A, while the epimer of iturin A shows a decrease in lipophilicity. It is important to note that this trend was consistent throughout the HPLC purification and analysis of the lipopeptides and with the different synthetic methods (liquid phase and on‐resin cyclization reactions).

**Figure 6:**
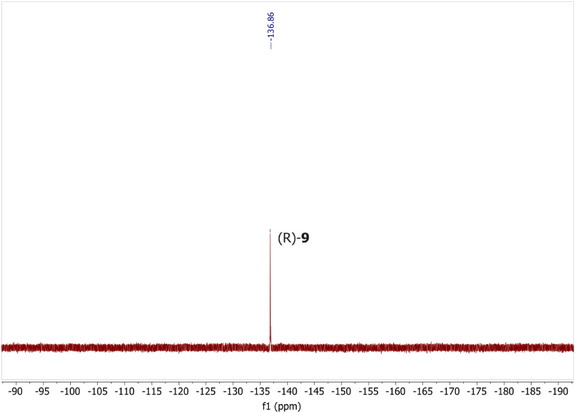
^19^F NMR spectrum of monofluorinated iturin A (*R*)‐**9** (564 MHz, 9:1 H_2_O:D_2_O).

Lastly, the ^19^F NMR spectrum of monofluorinated iturin A [(*R*)‐**9**] was obtained in aqueous conditions (Figure 6) at a concentration of 0.1 mM, showcasing its potential use as a mechanistic ^19^F NMR probe. Iturin A is poorly soluble in water,^[^ ^48^ ^]^ and this has hindered the NMR analysis of the lipopeptide under physiological conditions. In fact, NMR spectra of iturinic lipopeptides are only reported in d_6_‐DMSO solutions.^[^ ^16^, ^49^ ^]^ Future efforts should focus on exploiting the advantages of ^19^F NMR, such as its broad chemical shift range and negligible level of background signals, to study the mode of binding of the lipopeptide with cell membranes and to gain further insight into its mechanism of action.

### 2.3 Evaluation of Antifungal Activity

The antifungal activity of the obtained lipopeptides was evaluated by calculating their Minimum Inhibitory Concentration (MIC) against *Fusarium graminearum* and *Candida albicans*. *F. graminearum* is a filamentous fungus that causes Fusarium head blight, a devastating cereal crop disease that leads to reduced grain yield and quality.^[^ ^50^ ^]^ It also produces mycotoxins that are hazardous to human health.^[^ ^51^ ^]^ *C. albicans* is a yeast that acts as an opportunistic fungal pathogen and is responsible for about 70% of fungal infections around the world, some of which can lead to life‐threatening outcomes.^[^ ^52^ ^]^ The MIC was calculated via the broth microdilution method, and the results are presented in Table 1.

**Table 1:** MIC calculation for the three synthetic lipopeptides against *F. graminearum* and *C. albicans*. Commercially available iturin A was used as the positive control. The assays were performed in triplicate.

	MIC [µM]	
Peptides	*F. graminearum*	*C. albicans*
Commercial iturin A	32	64
(*R*)‐**8**: Synthetic iturin A_2_	16	64
(*S*)‐**8**: Epimer of iturin A_2_	>1024	>1024
(*R*)‐**9**: Monofluorinated iturin A_2_	16	64

The synthetic iturin A_2_ (*R*)‐**8** showed MIC values similar to the commercially available lipopeptide (Merck). The 2‐fold decrease in the MIC value against *F. graminearum* can be attributed to the various isoforms of iturin A (Figure 1) present in the commercially available bacterially isolated lipopeptide, indicating slightly different activities for different isoforms, as reported in the literature.^[^ ^53^ ^]^ Interestingly, the epimer of iturin A_2_, (*S*)‐**8**, lost all of its bioactivity under the conditions tested. This, to the best of our knowledge, is the first time that the dramatic importance of the stereochemistry of iturinic acid for the antifungal action of the natural lipopeptide has been demonstrated. It might also explain why this stereochemistry is strictly conserved among all the lipopeptides of the iturin family.^[^ ^6^ ^]^

The monofluorinated analog of iturin A_2_, (*R*)‐**9**, retained the activity of the natural lipopeptide. This is consistent with the results of previous bioactivity assays, where small amounts of fluorinated iturin A were obtained through a precursor directed biosynthesis approach.^[^ ^53^, ^54^ ^]^ The mechanism of interaction of Iturin A with the sterol of the fungal membrane has not been completely elucidated yet, but it is thought to involve hydrogen bond formation between the phenol ring and the fungal membrane sterols, with the hydroxy group of the phenol acting as a donor.^[^ ^16^ ^]^ The presence of the fluorine atom at the ortho position on the D‐Tyr ring makes the aromatic ring electron deficient, through its strong electron‐withdrawing inductive effect. Therefore, we reasoned that the polarised hydroxy group could show an enhanced hydrogen bond donation capacity, while the fluorine atom could act as a weak hydrogen bond acceptor.^[^ ^55^, ^56^ ^]^ The lower p*K*_a_ of the fluorinated tyrosine (p*K*_a_ = 8.8) in comparison to tyrosine (p*K*_a_ = 10.0) has been verified experimentally,^[^ ^57^ ^]^ but the phenol should be prevalently in its protonated form in the conditions used for the antifungal assays (pH = 7). Furthermore, the steric changes induced by the fluorine atom are expected to be minimal.^[^ ^58^ ^]^ However, our results show that monofluorination at the ortho position of the D‐Tyr residue did not have a measurable effect on the antifungal activity of the lipopeptide. Nevertheless, this novel Iturin A analog could be useful as a ^19^F NMR probe, for elucidation of the mode of binding of the natural lipopeptide to fungal membranes, given that it retains the bioactivity of the parent compound and given the suggested crucial role of the D‐Tyr residue on iturin A's mechanism of action.^[^ ^18^, ^33^ ^]^

Our results also highlight the need for further elucidation of the nature of the hydrogen bond formation between the lipopeptide and the compounds present in the fungal membrane. This can be achieved by the synthesis of further analogs of iturin A (i.e., analogs containing a difluorinated tyrosine residue) using the developed synthetic route.

Finally, we tested the presence of potential synergies between the here synthesised Iturin A lipopeptides and surfactin, against *F. graminearum* and *C. albicans*. Surfactin is a member of another class of cyclic lipopeptides produced by *Bacillus subtilis* and is considered an excellent biosurfactant while also possessing moderate antifungal activity.^[^ ^14^ ^]^ Synergistic effects between iturin A and surfactin concerning haemolysis,^[^ ^22^ ^]^ surface‐active properties,^[^ ^23^ ^]^ and antifungal activity against *Saccharomyces cerevisiae* ^[^ ^24^ ^]^ have been previously reported.

Surfactin alone did not show a pronounced antifungal effect, while weak synergistic effects were observed against *C. albicans*, at a 1:1 molar ratio of iturin A variants/surfactin (Table S1). In fact, we found that in combination with surfactin, the concentrations of both natural iturin A_2_ (*R*)‐**8** and of the monofluorinated analog (*R*)‐**9** needed for minimal inhibition decreased from 64 to 32 µM.

Further studies will be needed to determine the extent of synergistic effects for these lipopeptides at different ratios.

## 3 Conclusion

In this work, we reported a novel and efficient method for the total synthesis of the antifungal cyclic lipopeptide iturin A. This process included the enantioselective synthesis of iturinic acid with the use of a homochiral lithium amide, followed by Fmoc‐SPPS with an on‐resin cyclization step. The synthetic iturin A showed identical antimicrobial bioactivity to the commercially available lipopeptide against *Candida albicans* and a two‐fold increase in bioactivity against *Fusarium graminearum*.

Furthermore, this robust synthetic route was used to synthesise two novel iturin A analogs, to perform structure‐activity relationship studies regarding the importance of the stereochemistry of the β‐amino fatty acid and the impact of fluorination on bioactivity. We proved that the (*R*)‐configuration is essential for the antifungal activity of iturin A, while monofluorination did not alter the bioactivity. A ^19^F NMR spectrum of the monofluorinated iturin A analog was obtained in water for the first time, proving its suitability for ^19^F NMR studies in physiological conditions.

We anticipate that the developed synthesis will pave the way for accessing libraries of iturinic lipopeptide analogs that will shed light on structure structure‐activity relationship and mechanism of action of this extremely promising natural antimicrobial peptide.

## Supporting Information

The authors have cited additional references within the Supporting Information.^[^ ^59^, ^60^, ^61^, ^62^, ^63^ ^]^

## Conflict of Interest

The authors declare no conflict of interest.

## Supporting information



Supporting Information

## Data Availability

The data that support the findings of this study are available in the supplementary material of this article.

## References

[1] J. A. Hendrickson , C. Hu , S. L. Aitken , N. Beyda , Curr. Infect. Dis. Rep. 2019, 21, 47. 31734730 10.1007/s11908-019-0702-9

[2] M. C. Fisher , A. Alastruey‐Izquierdo , J. Berman , T. Bicanic , E. M. Bignell , P. Bowyer , M. Bromley , R. Brüggemann , G. Garber , O. A. Cornely , S. J. Gurr , T. S. Harrison , E. Kuijper , J. Rhodes , D. C. Sheppard , A. Warris , P. L. White , J. Xu , B. Zwaan , P. E. Verweij , Nat. Rev. Microbiol. 2022, 20, 557. 35352028 10.1038/s41579-022-00720-1 PMC8962932

[3] G. K. K. Reddy , A. R. Padmavathi , Y. V. Nancharaiah , Curr. Res. Microb. Sci. 2022, 3, 100137. 35909631 10.1016/j.crmicr.2022.100137 PMC9325902

[4] K. Nagórska , M. Bikowski , M. Obuchowski , Acta Biochim. Pol. 2007, 54, 495. 17882321

[5] T. Stein , Mol. Microbiol. 2005, 56, 845. 15853875 10.1111/j.1365-2958.2005.04587.x

[6] C. A. Dunlap , M. J. Bowman , A. P. Rooney , Front. Microbiol. 2019, 10, 1794. 31440222 10.3389/fmicb.2019.01794 PMC6693446

[7] D. Han , Y. Ji , S. Yang , P. Song , Y. Shi , D. Shao , X. Chen , L. Shang , J. Shi , C. Jiang , Antimicrob. Agents Chemother. 2024, 68, e0094823. 38051047 10.1128/aac.00948-23 PMC10777857

[8] M. Hua , Q. Deng , M. Qiu , Y. Deng , L. Sun , Z. Fang , J. Liao , J. Zhao , R. Gooneratne , Foods 2023, 12, 1278. 36981204 10.3390/foods12061278 PMC10048737

[9] J. Xiao , X. Guo , X. Qiao , X. Zhang , X. Chen , D. Zhang , Front. Microbiol. 2021, 12, 682437. 34220767 10.3389/fmicb.2021.682437 PMC8250863

[10] E. V. Shekunov , P. D. Zlodeeva , S. S. Efimova , A. A. Muryleva , V. V. Zarubaev , A. V. Slita , O. S. Ostroumova , Antiviral Res. 2023, 212, 105575. 36868316 10.1016/j.antiviral.2023.105575 PMC9977712

[11] R. K. T. D. Sebastian , Biocatal. Agric. Biotechnol. 2021, 36, 102125.

[12] G. Dey , R. Bharti , G. Dhanarajan , S. Das , K. K. Dey , B. N. P. Kumar , R. Sen , M. Mandal , Sci. Rep. 2015, 5, 10316. 25974307 10.1038/srep10316 PMC4431395

[13] D. A. Yaraguppi , Z. K. Bagewadi , N. R. Patil , N. Mantri , Biomolecules 2023, 13, 1515. 37892197 10.3390/biom13101515 PMC10604914

[14] M. Ongena , P. Jacques , Trends Microbiol. 2008, 16, 115. 18289856 10.1016/j.tim.2007.12.009

[15] U. Nagai , F. Besson , F. Peypoux , Tetrahedron Lett. 1979, 20, 2359.

[16] L. Volpon , F. Besson , J. Lancelin , Eur. J. Biochem. 1999, 264, 200. 10447689 10.1046/j.1432-1327.1999.00605.x

[17] R. Maget‐Dana , F. Peypoux , Toxicology 1994, 87, 151. 8160184 10.1016/0300-483x(94)90159-7

[18] R. Maget‐Dana , M. Ptak , F. Peypoux , G. Michel , Biochim. Biophys. Acta 1987, 898, 1. 3828330 10.1016/0005-2736(87)90104-0

[19] J. M. Bland , J. Org. Chem. 1996, 61, 5663.

[20] S. Lei , H. Zhao , B. Pang , R. Qu , Z. Lian , C. Jiang , D. Shao , Q. Huang , M. Jin , J. Shi , Appl. Microbiol. Biotechnol. 2019, 103, 4377. 30997554 10.1007/s00253-019-09805-z

[21] Y. Wang , C. Zhang , J. Liang , L. Wu , W. Gao , J. Jiang , Front. Microbiol. 2020, 11, 536083. 33013776 10.3389/fmicb.2020.536083 PMC7509112

[22] R. Maget‐Dana , L. Thimon , F. Peypoux , M. Ptak , Biochimie 1992, 74, 1047. 1292612 10.1016/0300-9084(92)90002-v

[23] H. Razafindralambo , Y. Popineau , M. Deleu , C. Hbid , P. Jacques , P. Thonart , M. Paquot , Langmuir 1997, 13, 6026.

[24] L. Thimon , F. Peypoux , R. Dana Maget , B. Roux , G. Michel , Biotechnol. Appl. Biochem. 1992, 16, 144. 1457050

[25] S. L. Puan , P. Erriah , M. M. A. Baharudin , N. M. Yahaya , W. N. I. W. A. Kamil , M. S. M. Ali , S. A. Ahmad , S. N. Oslan , S. Lim , S. Sabri , Appl. Microbiol. Biotechnol. 2023, 107, 5569. 37450018 10.1007/s00253-023-12651-9

[26] I. M. Banat , Q. Carboué , G. Saucedo‐Castañeda , J. de Jesús Cázares‐Marinero , Bioresour. Technol. 2021, 320, 124222. 33171346 10.1016/j.biortech.2020.124222

[27] Y. Xu , D. Cai , H. Zhang , L. Gao , Y. Yang , J. Gao , Y. Li , C. Yang , Z. Ji , J. Yu , S. Chen , Process Biochem. 2020, 90, 50.

[28] V. V. Yim , I. Kavianinia , A. J. Cameron , P. W. R. Harris , M. A. Brimble , Org. Biomol. Chem. 2020, 18, 2838. 32048704 10.1039/d0ob00203h

[29] P. Karamanis , J. Muldoon , C. D. Murphy , M. Rubini , J. Pept. Sci. 2024, 30, e3569. 38301277 10.1002/psc.3569

[30] J. M. Palomo , RSC Adv. 2014, 4, 32658.

[31] A. A. Berger , J. Völler , N. Budisa , B. Koksch , Acc. Chem. Res. 2017, 50, 2093. 28803466 10.1021/acs.accounts.7b00226

[32] S. L. Cobb , C. D. Murphy , J. Fluorine Chem. 2009, 130, 132.

[33] D. Gimenez , A. Phelan , C. D. Murphy , S. L. Cobb , Beilstein J. Org. Chem. 2021, 17, 293. 33564338 10.3762/bjoc.17.28 PMC7849273

[34] J. M. Bland , Synth. Commun. 1995, 25, 467.

[35] M. Liu , M. P. Sibi , Tetrahedron 2002, 58, 7991.

[36] M. P. Sibi , P. K. Deshpande , J. Chem. Soc., Perkin Trans. 2000, 1, 1461.

[37] S. G. Davies , O. Ichihara , Tetrahedron: Asymmetry 1991, 2, 183.

[38] S. G. Davies , A. D. Smith , P. D. Price , Tetrahedron: Asymmetry 2005, 16, 2833.

[39] D. Roman , M. Sauer , C. Beemelmanns , Synthesis 2021, 53, 2713.

[40] M. Einsiedler , C. S. Jamieson , M. A. Maskeri , K. N. Houk , T. A. M. Gulder , Angew. Chem., Int. Ed. 2021, 60, 8297. 10.1002/anie.202017086 PMC8049060 33411393

[41] D. Gimenez , A. Phelan , C. D. Murphy , S. L. Cobb , Org. Lett. 2021, 23, 4672. 34077216 10.1021/acs.orglett.1c01387 PMC8289291

[42] M. Obkircher , C. Stähelin , F. Dick , J. Pept. Sci. 2008, 14, 763. 18219706 10.1002/psc.1001

[43] C. Bechtler , C. Lamers , RSC Med. Chem. 2021, 12, 1325. 34447937 10.1039/d1md00083g PMC8372203

[44] F. Albericio , J. M. Bofill , A. El‐Faham , S. A. Kates , J. Org. Chem. 1998, 63, 9678.

[45] J. A. K. Twibanire , T. B. Grindley , Org. Lett. 2011, 13, 2988. 21591807 10.1021/ol201005s

[46] T. Conroy , K. A. Jolliffe , R. J. Payne , Org. Biomol. Chem. 2009, 7, 2255. 19462031 10.1039/b821051a

[47] J. Gregorc , N. Lensen , G. Chaume , J. Iskra , T. Brigaud , J. Org. Chem. 2023, 88, 13169. 37672679 10.1021/acs.joc.3c01373 PMC10507666

[48] F. Besson , C. Raimbault , M. Hourdou , R. Buchet , Spectrochim. Acta, Part A 1996, 52, 793.

[49] C. Garbay‐Jaureguiberry , B. Roques , L. Delcambe , F. Peypoux , G. Michel , FEBS Lett. 1978, 93, 151. 100346 10.1016/0014-5793(78)80825-4

[50] L. E. Osborne , J. M. Stein , Int. J. Food Microbiol. 2007, 119, 103. 17716761 10.1016/j.ijfoodmicro.2007.07.032

[51] Y. Chen , H. C. Kistler , Z. Ma , Annu. Rev. Phytopathol. 2019, 57, 15. 30893009 10.1146/annurev-phyto-082718-100318

[52] J. Talapko , M. Juzbašić , T. Matijević , E. Pustijanac , S. Bekic , I. Kotris , I. Škrlec , J. Fungi. (Basel) 2021, 7, 79. 33499276 10.3390/jof7020079 PMC7912069

[53] N. K. O'Connor , A. S. Hudson , S. L. Cobb , D. O'Neil , J. Robertson , V. Duncan , C. D. Murphy , Amino Acids 2014, 46, 2745. 25193167 10.1007/s00726-014-1830-z

[54] S. Moran , D. K. Rai , B. R. Clark , C. D. Murphy , Org. Biomol. Chem. 2009, 7, 644. 19194576 10.1039/b816345f

[55] G. Xiao , J. F. Parsons , K. Tesh , R. N. Armstrong , G. L. Gilliland , J. Mol. Biol. 1998, 281, 323. 9698551 10.1006/jmbi.1998.1935

[56] P. P. Pal , J. H. Bae , M. K. Azim , P. Hess , R. Friedrich , R. Huber , L. Moroder , N. Budisa , Biochemistry 2005, 44, 3663. 15751943 10.1021/bi0484825

[57] J. S. Thorson , E. Chapman , E. C. Murphy , P. G. Schultz , J. K. Judice , J. Am. Chem. Soc. 1995, 117, 1157.

[58] D. B. Harper , D. O'Hagan , Nat. Prod. Rep. 1994, 11, 123. 15209126 10.1039/np9941100123

[59] L. Corcilius , D. Y. Liu , J. L. Ochoa , R. G. Linington , R. J. Payne , Org. Biomol. Chem. 2018, 16, 5310. 29993080 10.1039/c8ob01268g

[60] J. Monfray , Y. Gelas‐Mialhe , J. Gramain , R. Remuson , Tetrahedron Lett. 2003, 44, 5785.

[61] E. Kaiser , R. Colescott , C. Bossinger , P. Cook , Anal. Biochem. 1970, 34, 595. 5443684 10.1016/0003-2697(70)90146-6

[62] Clinical and Laboratory Standards Institute . Reference Method for Broth Dilution Antifungal Susceptibility Testing of Yeasts; Approved Standard – second edition. CLSI document M27‐A2, Wayne, Pa., 2002.

[63] Clinical and Laboratory Standards Institute . Reference Method for Broth Dilution Antifungal Susceptibility Testing of Filamentous Fungi; Approved Standard – first edition. CLSI document M38‐A, Wayne, Pa., 2002.

