# Supplementary material for: Novel Synthesis of the Antifungal Cyclic Lipopeptide Iturin A and Its Fluorinated Analog for Structure‐Activity Relationship Studies
Source: Chemistry. 2025 Jul 25;31(47):e01341. doi: 10.1002/chem.202501341 (PMC12376246; doi:10.1002/chem.202501341)
Supplement: Supplementary file 1 — Supporting Information [file CHEM-31-e01341-s001.pdf]

Supporting Information

Novel Synthesis of the Antifungal Cyclic Lipopeptide Iturin A and its Fluorinated Analogue for Structure-activity Relationship studies

Periklis Karamanis[a],[b], Matthew Kiernan[a],[b], Jimmy Muldoon[a], Finn Doyle[a], Paul Evans[a],[b], Cormac D. Murphy*[b],[c],[d], and Marina Rubini*[a],[b]

[a] P. Karamanis, M. Kiernan, J. Muldoon, F. Doyle, P. Evans, M. Rubini

School of Chemistry, University College Dublin, Dublin, Ireland

E-mail: marina.rubini@ucd.ie

[b] P. Karamanis, M. Kiernan, P. Evans, C.D. Murphy, M. Rubini

BiOrbic Bioeconomy SFI Research Centre, University College Dublin, Dublin, Ireland

[c] Cormac D. Murphy

School of Biomolecular and Biomedical Science, University College Dublin, Dublin, Ireland

E-mail: cormac.d.murphy@ucd.ie

[d] Cormac D. Murphy

Conway Institute for Biomolecular and Biomedical Research, University College Dublin, Dublin, Ireland

Contents

General methods S3

Synthesis of compound (*R*)-7 .... S4

Ethyl (*E*)-tetradec-2-enoate (2): S4

Ethyl (*R*)-3-(benzyl((*R*)-1-phenylethyl)amino)tetradecanoate ((*R*,*R*)-3): S4

Ethyl (*R*)-3-((*tert*-butoxycarbonyl)amino)tetradecanoate ((*R*)-4): S5

(*R*)-3-((*tert*-Butoxycarbonyl)amino)tetradecanoic acid ((*R*)-5)): S5

Benzyl (*R*)-3-((*tert*-butoxycarbonyl)amino)tetradecanoate ((*R*)-6): S6

Figure S1: Chiral HPLC analysis of (A) the 1:1 mixture of **(*R*)-6** and **(*S*)-6** and (B) purified **(*R*)-6** S7

(*R*)-3-((((9*H*-fluoren-9-yl)methoxy)carbonyl)amino)tetradecanoic acid ((*R*)-7): S7

Synthesis of compound (*S*)-7: S7

Ethyl (*S*)-3-(benzyl((*S*)-1-phenylethyl)amino)tetradecanoate ((*S*,*S*)-3): S7

Ethyl (*S*)-3-((*tert*-butoxycarbonyl)amino)tetradecanoate ((*S*)-4): S8

(*S*)-3-((*tert*-Butoxycarbonyl)amino)tetradecanoic acid ((*S*)-5)): S8

Benzyl (*S*)-3-((*tert*-butoxycarbonyl)amino)tetradecanoate ((*S*)-6): S9

Figure S2: Chiral HPLC analysis of (A) the 1:1 mixture of **(*R*)-6** and **(*S*)-6** and (B) purified **(*S*)-6** S9

(*S*)-3-((((9*H*-fluoren-9-yl)methoxy)carbonyl)amino)tetradecanoic acid ((*S*)-7): S9

Solid-phase Peptide Synthesis and cyclisation of (*R*)-8, (*S*)-8, and (*R*)-9: S10

Method A – SPPS with the cyclisation in liquid-phase: S10

Method B – SPPS with the cyclisation on resin: S11

Figure S3: Comparison between the crude HPLC chromatogram of **(*R*)-9** after its synthesis with (A) method A and (B) method B. S12

Antifungal susceptibility testing S13

Table S1: MIC calculation for surfactin and for the obtained bioactive lipopeptides in the presence of surfactin (1:1) iturin A / surfactin ratio) against *F. graminearum* and *C. albicans*. S13

^{1}H, ^{13}C and ^{19}F NMR Spectra S14

References S19

General methods

All protected amino acids (95% purity or higher) were purchased from Iris Biotech GMBH (Marktredwitz, Germany). Iturin A (≥95%) was purchased from Merck KGaA (Darmstadt, Germany) as a mixture of isoforms. Solvents and reagents (reagent grade or better) were purchased from Merck KGaA. Chemical reactions were monitored using analytical thin-layer chromatography, performed using aluminium-backed silica plates (60 F254) and the stated eluents. Visualization was accomplished using ultraviolet light and/or a potassium permanganate stain. Product purification by flash column chromatography was performed using silica gel (Davisil, 230–400 mesh, 40–63 µm).

^{1}H NMR spectra were recorded using a Varian VnmrS (400 MHz) spectrometer. Samples were dissolved in $CDCl_3$ and referenced to TMS (0.00 ppm). ^{13}C NMR spectra were recorded using a Varian VnmrS (100 MHz) spectrometer. Samples were dissolved in $CDCl_3$ and referenced to TMS (0.00 ppm). ^{19}F NMR spectra were recorded using a Varian VnmrS (376 MHz) or a Varian VnmrS (564 MHz) spectrometer. Samples were dissolved in CD_3OD or in 9:1 H_2O:D_2O and referenced to Trifluoroacetic acid (-77.00 ppm). Spectra were analysed using MestreNova 14.0. Chemical shifts are reported in parts per million (ppm) and coupling constants (J) are given in Hertz. Multiplicities are abbreviated as s (singlet), d (doublet), t (triplet), q (quartet), m (multiplet) or combinations thereof.

Cyclic lipopeptides were purified using an Agilent 1260 high-performance liquid chromatography system with a C_{18} column (Agilent Zorbax SB-C_{18}, 9.4x250 mm) using a binary solvent system with a linear gradient of acetonitrile and water (both containing 0.1% TFA), changing over 40 min from 10%–100% acetonitrile, followed by a final isocratic hold for 5 min. The flow rate was set at 4 mL/min with UV detection at 220 nm. Chiral HPLC analysis was performed using an Agilent 1260 HPLC with a Daicel Chiralpak IA column (4.6 x 250 mm) using a binary solvent system with an isocratic gradient of hexane: isopropanol (98:2) for 90 minutes. The flow rate was set at 0.25 mL/min with UV detection at 210 nm.

LC–MS data were acquired in ESI+ mode on an Agilent 6546 QToF system equipped with an Agilent JetStream (AJS) ESI source and coupled with an Agilent 1260 Infinity Prime II LC system. Chromatography was carried out with a C_{18} column (Agilent Zorbax Eclipse Plus, 2.1 × 50 mm) using a binary solvent system with a linear gradient of acetonitrile and water (containing 0.1% formic acid) changing over 5 min from 10%–90% acetonitrile, followed by a final isocratic hold for 5 min. The flow rate was set at 0.6 mL/min. The QToF AJS ESI source was configured with a drying gas temperature of 325 °C, sheath gas temperature 350 °C, Capillary voltage 4000 V, nozzle voltage 2000 V, fragmentor voltage 175 V, skimmer voltage 65 V. Data were processed with the Agilent Masshunter software. Target compounds were searched via compound matching using the Agilent FBF (Find-By Formula) algorithm, matching for singly charged monomeric ion species for common ions such as $[M+H]^+$ and $[M+Na]^+$.

Abbreviations: Dmab = 4-{*N*-[1-(4,4-dimethyl-2,6-dioxocyclohexylidene)-3-methylbutyl]-amino} benzyl; Fmoc = 9-Fluorenylmethyloxycarbonyl; Fmoc-OSu = N-(9-Fluorenylmethoxycarbonyloxy)succinimide; TIPS = Triisopropylsilane; Trt = Trityl

Synthesis of compound (*R*)-7

Ethyl (*E*)-tetradec-2-enoate (2):

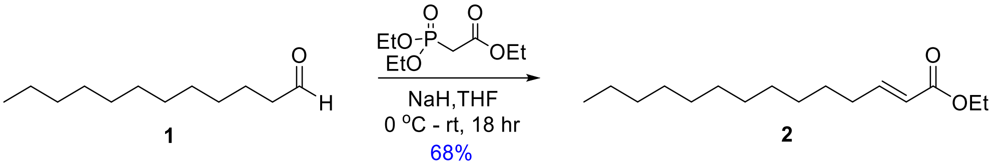

Under anhydrous conditions at 0 ˚C, sodium hydride (723 mg, 90% w/w dispersion in oil, 27.13 mmol, 2.50 equiv.) was slowly added to a solution of triethylphosphonoacetate (5.38 mL, 27.13 mmol, 2.50 equiv.) in anhydrous tetrahydrofuran (40 mL). The resulting slurry was stirred at 0 ˚C for a further thirty minutes before dodecanal (**1**, 2.41 mL, 2.00 g, 10.85 mmol, 1.00 equiv.) was added dropwise. This reaction mixture was allowed to warm up to room temperature and stirred for eighteen hours under anhydrous conditions. After quenching the reaction with water (20 mL), the aqueous layer was extracted with ethyl acetate (2 x 30 mL) and the combined organic layers were washed with brine (50 mL), dried over magnesium sulfate, filtered and concentrated *in vacuo*. The crude product was purified by flash chromatography (19:1 cyclohexane: ethyl acetate) to afford ethyl (*E*)-tetradec-2-enoate (**2**) as a colourless liquid (1.880 g, 7.40 mmol, 68%); **R_f** = 0.5 (19:1 cyclohexane:ethyl acetate); **^{1}H NMR** (400 MHz, $CDCl_3$): 6.94 (1H, dt, J = 15.5, 7.0 Hz, CH), 5.79 (1H, dt, J = 15.5, 2.0 Hz, CH), 4.17 (2H, q, J = 7.5 Hz, CH_2), 2.22-2.13 (2H, m, CH_2), 1.47-1.37 (2H, m, CH_2), 1.33-1.22 (19H, m, CH_2, CH_3), 0.86 (3H, t, J = 7.0 Hz, CH_3) ppm. Data consistent with literature.[1]

Ethyl (*R*)-3-(benzyl((*R*)-1-phenylethyl)amino)tetradecanoate ((*R*,*R*)-3):

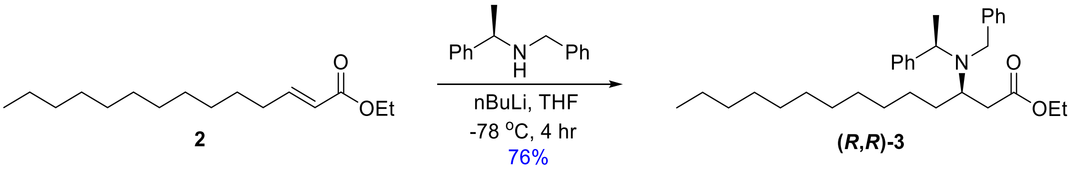

Under anhydrous conditions at -78 ˚C, *n*-butyllithium (5.54 mL, 2.50 M in hexane, 13.86 mmol, 2.00 equiv.) was added dropwise over ten minutes to a solution of (*R*)-*N*-benzyl-α-methylbenzylamine (2.90 mL, 13.86 mmol, 2.00 equiv.) in anhydrous tetrahydrofuran (20 mL). The resulting purple solution was stirred at -78 ˚C for a further thirty minutes before a solution of ethyl (*E*)-tetradec-2-enoate **2** (1.763 g, 6.93 mmol, 1.00 equiv.) in anhydrous tetrahydrofuran (2 mL) was added dropwise over ten minutes. This reaction mixture was stirred at -78 ˚C for four hours under anhydrous conditions. After quenching the reaction with saturated ammonium chloride solution (6 mL) and diluting with water (20 mL), the layers were separated. The mixture was extracted with diethyl ether (3 x 30 mL) and the combined organic layers were washed with brine (40 mL), dried over magnesium sulfate, filtered and concentrated *in vacuo*. The crude product was purified by flash chromatography (19:1 cyclohexane:ethyl acetate) to afford ethyl (*R*)-3-(benzyl((*R*)-1-phenylethyl)amino) tetradecanoate (**(*R*,*R*)-3**) as a colourless oil (2.455 g, 5.27 mmol, 76%); **R_f** = 0.3 (4:1 pentane: dichloromethane); **^{1}H NMR** (400 MHz, $CDCl_3$): δ = 7.42 (d, J = 6.8 Hz, 2H), 7.35-7.19 (m, 8H), 4.08-3.93 (m, 2H), 3.83 (q, J = 7.0 Hz, 1H), 3.79 (d, J = 15.0 Hz, 1H), 3.54 (d, J = 15.0 Hz, 1H), 3.30 (tt, J = 8.2, 4.7 Hz, 1H),

2.05 (dd, J = 14.5, 4.6 Hz, 1H), 1.99 (dd, J = 14.5, 8.4 Hz, 1H), 1.54-1.46 (m, 2H), 1.33 (d, J = 7.0 Hz, 3H), 1.30-1.22 (m(br), 18H), 1.18 (t, J = 7.1 Hz, 3H), 0.89 (t, J = 7.0 Hz, 3H) ppm; **^{13}C NMR** (100 MHz, $CDCl_3$): δ = 172.9 (C), 143.3 (C), 141.8 (C), 128.3 (2xCH), 128.2 (2xCH), 128.1 (2xCH), 127.9 (2xCH), 126.9 (CH), 126.6 (CH), 60.1 (CH_2), 58.0 (CH), 54.1 (CH), 50.0 (CH_2), 36.7 (CH_2), 33.5 (CH_2), 31.9 (CH_2), 29.7 (CH_2), 29.7 (3xCH_2), 29.6 (CH_2), 29.4 (CH_2), 27.0 (CH_2), 22.7 (CH_2), 19.7 (CH_3), 14.2 (CH_3), 14.1 (CH_3) ppm; **HRMS** (ESI-TOF) calculated for $C_{31}H_{47}NO_2$ 465.3607, found 466.3605; **IR** (neat): 2923.0, 2852.6, 1731.1, 1453.2, 1148.7, 1027.1, 746.6, 698.3 cm^{-1}; **[α]$_D^{21}$** = +5.9 (c = 1.02, $CHCl_3$). Data consistent with literature.[2]

Ethyl (*R*)-3-((*tert*-butoxycarbonyl)amino)tetradecanoate ((*R*)-4):

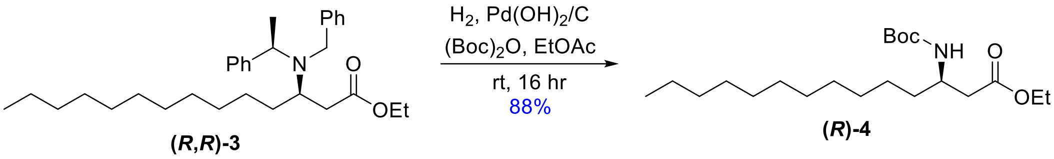

(*R*,*R*)-3 **(*R*)-4**

Under a hydrogen atmosphere (balloon), a solution of (*R*)-3-(benzyl((*R*)-1-phenylethyl)amino) tetradecanoate **(*R*,*R*)-3** (2.060 g, 4.42 mmol, 1.00 equiv.), palladium hydroxide (621 mg, 20% w/w on carbon, 0.88 mmol, 20 mol%) and di-*tert*-butyl decarbonate (1.159 g, 5.31 mmol, 1.20 equiv.) in ethyl acetate (35 mL) was stirred at room temperature for sixteen hours. The reaction mixture was filtered through celite and the filtrate was concentrated *in vacuo*. The crude product was purified by flash chromatography (19:1 to 9:1 cyclohexane:ethyl acetate) to afford ethyl (*R*)-3-((*tert*-butoxycarbonyl)amino)tetradecanoate (**(*R*)-4**) as a colourless liquid (1.445 g, 3.89 mmol, 88%); **R_f** = 0.2 (19:1 cyclohexane: ethyl acetate); **^{1}H NMR** (400 MHz, $CDCl_3$): 4.90 (d, J = 9.3 Hz, 1H), 4.14 (q, J = 7.1 Hz, 2H), 3.93-3.85 (m, 1H), 2.54-2.43 (m, 2H), 1.51-1.46 (m, 2H), 1.43 (s(br), 9H), 1.34-1.23 (m, 21H), 0.88 (t, J = 7.0 Hz, 3H) ppm; **^{13}C NMR** (100 MHz, $CDCl_3$): 171.7 (C), 155.3 (C), 79.1 (C), 60.4 (CH_2), 47.6 (CH), 39.3 (CH_2), 34.7 (CH_2), 31.7 (CH_2), 29.6 (CH_2), 29.6 (CH_2), 29.5 (CH_2), 29.5 (CH_2), 29.3 (CH_2), 29.3 (CH_2), 28.4 (3xCH_3), 26.1 (CH_2), 22.7 (CH_2), 14.2 (CH_3), 14.1 (CH_3) ppm; **HRMS** (ESI-TOF) calculated for $C_{21}H_{41}NO_4$ 371.3036, found 371.3037; **IR** (neat): 2978.3, 2924.7, 2854.4, 1810.7, 1715.7, 1501.3, 1367.8, 1167.7, 1115.9, 1066.5, 645.8 cm^{-1}; **[α]$_D^{21}$** = +12.7 (c = 1.02, $CHCl_3$).

(*R*)-3-((*tert*-Butoxycarbonyl)amino)tetradecanoic acid ((*R*)-5)):

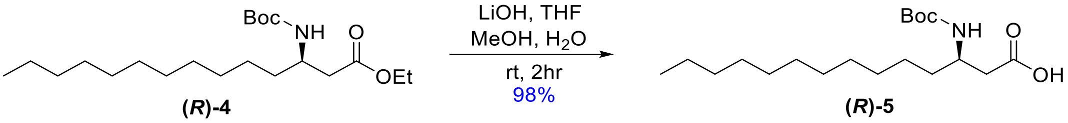

(*R*)-4 **(*R*)-5**

To a solution of ethyl (*R*)-3-((*tert*-butoxycarbonyl)amino)tetradecanoate **(*R*)-4** (1.389 g, 3.74 mmol, 1.00 equiv.) in tetrahydrofuran (37 mL) and methanol (11.5 mL) was added a solution of lithium hydroxide monohydrate (471 mg, 11.22 mmol, 3 equiv.) in water (11.5 mL). The resulting reaction mixture was stirred at room temperature for three hours before being diluted with ethyl acetate (50 mL) and washed with hydrochloric acid solution (1M, 40 mL). The aqueous layer was back extracted with ethyl acetate (2 x 50 mL) and the combined organic layers were washed with water (50 mL) and brine (60 mL), dried over magnesium sulfate,

filtered and concentrated *in vacuo* to afford (*R*)-3-((*tert*-butoxycarbonyl)amino)tetradecanoic acid (**(*R*)-5**)) as a white solid (1.261 g, 3.67 mmol, 98%); **R$_f$** = 0.1 with streaking (5:1 cyclohexane:ethyl acetate); **MP** = 62-64 ˚C; **^{1}H NMR** (400 MHz, $CDCl_3$): 4.93-4.83 (m(br), 1H), 3.95-3.81 (m(br), 1H), 2.63-2.44 (m(br), 2H), 1.56-1.49 (m, 2H), 1.44 (s, 9H), 1.36-1.20 (m(br), 18H), 0.88 (t, *J* = 7.1 Hz, 3H) ppm; **^{13}C NMR** (100 MHz, $CDCl_3$): 176.3 (C), 146.6 (C), 79.5 (C), 47.5 (CH), 39.2 (CH_2), 34.6 (CH_2), 31.9 (CH_2), 29.6 (CH_2), 29.6 (CH_2), 29.5 (CH_2), 29.5 (CH_2), 29.3 (2xCH_2), 28.4 (3xCH_3), 26.1 (CH_2), 22.7 (CH_2), 14.1 (CH_3) ppm; **HRMS** (ESI-TOF) calculated for $C_{19}H_{37}NO_4$ 343.2723, found 344.2724; **IR** (neat): 3352.3, 2915.5, 2849.5, 1690.8, 1524.7, 1446.2, 1365.9, 1274.8, 1171.7, 1065.8, 649.4 cm^{-1}. **$[\alpha]_D^{21}$** = +6.3 (*c* = 0.32, MeOH); lit. **$[\alpha]_D^{20}$** +5.3 (*c* = 0.9, MeOH). Data is consistent with literature.[3] The enantiopurity of **(*R*)-5** was determined by converting it to the corresponding benzyl ester **(*R*)-6** and then subjecting it to chiral-HPLC analysis (see below).

Benzyl (*R*)-3-((*tert*-butoxycarbonyl)amino)tetradecanoate ((*R*)-6):

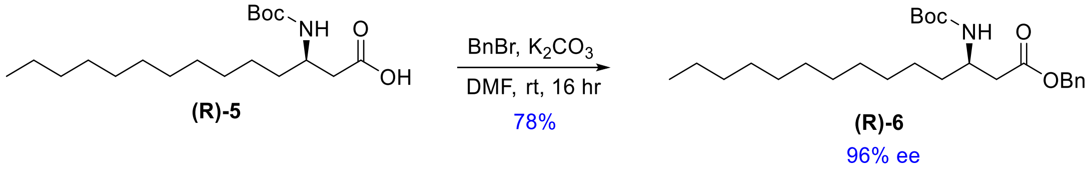

To a solution of (*R*)-3-((*tert*-butoxycarbonyl)amino)tetradecanoic acid **(*R*)-5** (70 mg, 0.20 mmol, 1.00 equiv.) in dimethylformamide (2 mL) was added potassium carbonate (56.3 mg, 0.41 mmol, 2.00 equiv.). Benzyl bromide (29 µL, 41.8 mg, 0.24 mmol, 1.20 equiv.) was then added and the resulting reaction mixture was stirred at room temperature for sixteen hours. The reaction mixture was diluted with water (10 mL) and the product was extracted with dichloromethane (3 x 10 mL). The combined organic layers were washed with brine (15 mL), dried over magnesium sulfate, filtered and concentrated *in vacuo*. The crude product was purified by flash chromatography (6:1 cyclohexane: ethyl acetate) to afford benzyl (*R*)-3-((*tert*-butoxycarbonyl) amino)tetradecanoate (**(*R*)-6**) as a white solid (69.1 mg, 0.16 mmol, 78%); **R$_f$** = 0.4 (6:1 cyclohexane: ethyl acetate). Characterisation data is consistent with the literature.[3]

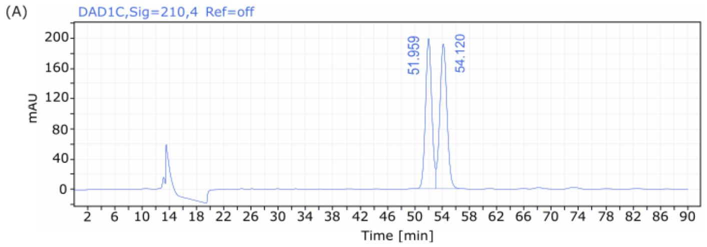

Signal: DAD1C,Sig=210,4 Ref=off

RT [min]	Type	Width [min]	Area	Height	Area%
51.959	BV	2.58	12709.18	198.58	47.83
54.120	VB	3.47	13865.12	191.52	52.17
		Sum	26574.29		

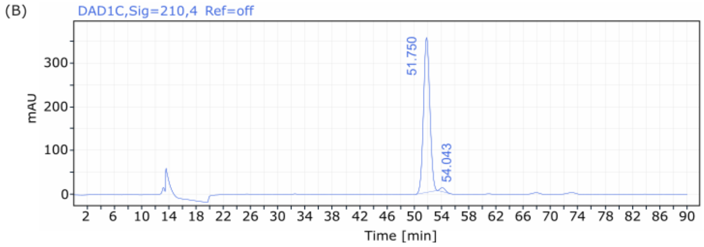

Signal: DAD1C,Sig=210,4 Ref=off

RT [min]	Type	Width [min]	Area	Height	Area%
51.750	BB	3.22	22882.79	353.43	97.91
54.043	BB	1.90	489.37	9.04	2.09
		Sum	23372.17		

Figure S1: Chiral HPLC analysis of (A) the 1:1 mixture of ***(R)*-6** and ***(S)*-6** and (B) purified ***(R)*-6**, t_1 = 51.750 min (minor), t_2 = 54.042 min (major). Daicel Chiralpak IA column, 98:2 hexane: isopropanol, 0.25 mL/min, 210 nm.

(*R*)-3-((((9*H*-fluoren-9-yl)methoxy)carbonyl)amino)tetradecanoic acid ((*R*)-7):

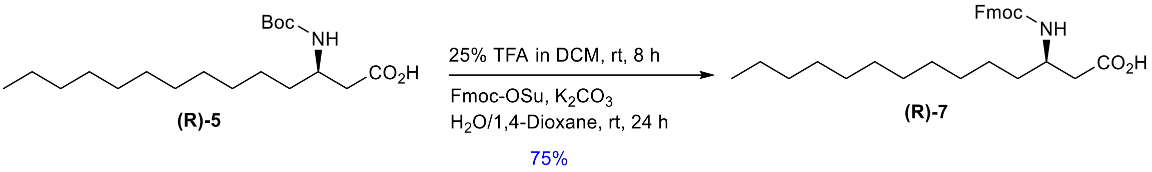

(*R*)-3-((*tert*-Butoxycarbonyl)amino)tetradecanoic acid ***(R)*-5** (500 mg, 1.46 mmol, 1.00 equiv.) was dissolved in dichloromethane (DCM) containing 25% v/v trifluoroacetic acid and the mixture was stirred at room temperature for 8 hours. The solvent was then removed under reduced pressure and the resulting white solid was dissolved in 10 mL of H_2O:1,4-dioxane (1:1). Potassium carbonate (605 mg, 4,38 mmol, 3.00 equiv.) was added and the mixture was cooled to 0 ºC. A solution of Fmoc-OSu (739 mg, 2.19 mmol, 1.50 equiv.) in 1,4-dioxane (5 mL) was added dropwise and the mixture was stirred for 24 hours. The solvent was removed under reduced pressure and H_2O (20 mL) was added. The solution was acidified with a 5 M solution of hydrochloric acid (pH = 2) and extracted with ethyl acetate (3 x 30 mL). The combined organic layers were washed with brine (30 mL) and dried over anhydrous sodium sulfate. After filtration the solvent was removed under reduced pressure and the crude product was purified by flash chromatography (9:1 DCM:MeOH) to afford the desired product as a white solid (510 mg, 1.095 mmol, 75%). $\mathbf{R_f}$ = 0.5 with streaking (9:1 DCM:MeOH); ^{1}**H NMR** (400 MHz, $CDCl_3$): 7.74 (d, J = 7.5 Hz, 2H), 7.57 (d, J = 7.5 Hz, 2H), 7.38 (t, J = 7.5 Hz, 2H), 7.29 (td, J = 7.5, 1.1 Hz, 2H), 5.11 (d, J = 8.8 Hz, 1H), 4.39 (d, J = 6.5 Hz, 2H), 4.20 (t, J = 6.5 Hz, 1H), 3.99-3.92 (m(br), 1H), 2.63-2.52 (m, 2H), 1.57-1.51 (m(br), 2H), 1.35-1.17 (m(br), 18H), 0.87 (t, J = 6.7 Hz, 3H) ppm; ^{13}**C NMR** (100 MHz, $CDCl_3$): 176.3 (C), 155.9 (C), 143.9 (2xC), 141.3 (2xC), 127.7 (2xCH), 127.0 (2xCH), 125.0 (2xCH), 120.0 (2xCH), 66.6 (CH_2), 48.0 (CH), 47.2 (CH), 38.9 (CH_2), 34.3 (CH_2), 31.9 (CH_2), 29.6 (CH_2), 29.6 (CH_2), 29.5 (CH_2), 29.5 (CH_2), 29.3 (CH_2), 29.3 (CH_2), 26.1 (CH_2), 22.7 (CH_2), 14.1 (CH_3) ppm. **HRMS** (ESI-TOF) calculated for $C_{29}H_{40}NO_4^+$ $[M+H]^+$ 466.2952, found 466.2954.

Synthesis of compound (*S*)-7:

Ethyl (*S*)-3-(benzyl((*S*)-1-phenylethyl)amino)tetradecanoate ((*S*,*S*)-3):

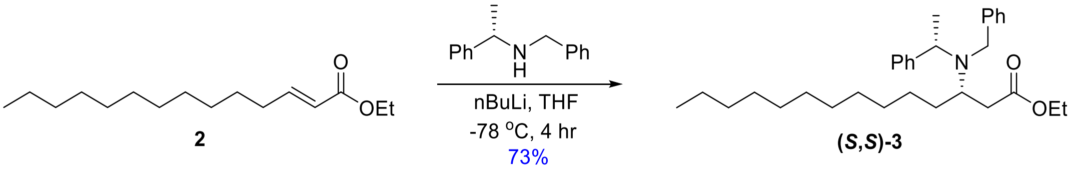

Under anhydrous conditions at -78 ˚C, *n*-butyllithium (3.77 mL, 2.50 M in hexane, 9.43 mmol, 2.00 equiv.) was added dropwise over ten minutes to a solution of (*S*)-*N*-benzyl-α-methylbenzylamine (1.97 mL, 9.43 mmol, 2.00 equiv.) in anhydrous tetrahydrofuran (14 mL). The resulting purple solution was stirred at -78 ˚C for a further thirty minutes before a solution of ethyl (*E*)-tetradec-2-enoate **2** (1.200 g, 4.72 mmol, 1.00 equiv.) in anhydrous tetrahydrofuran

(2 mL) was added dropwise over ten minutes. This reaction mixture was stirred at -78 ˚C for four hours under anhydrous conditions. The reaction was quenched with saturated ammonium chloride solution (4 mL) and diluted with water (15 mL).The mixture was then extracted with diethyl ether (3 x 20 mL). The combined organic layers were washed with brine (30 mL), dried over magnesium sulfate, filtered and concentrated *in vacuo*. The crude product was purified by flash chromatography (19:1 cyclohexane:ethyl acetate) to afford ethyl (*S*)-3-(benzyl((*S*)-1-phenylethyl)amino) tetradecanoate (**(*S*,*S*)-3**) as a colourless oil (1.612 g, 3.45 mmol, 73%); **[α]$_D$^{21}** = -5.8 (*c* = 1.04, $CHCl_3$); All other characterisation matched that of (***R*,*R*)-3**.

Ethyl (*S*)-3-((*tert*-butoxycarbonyl)amino)tetradecanoate ((*S*)-4):

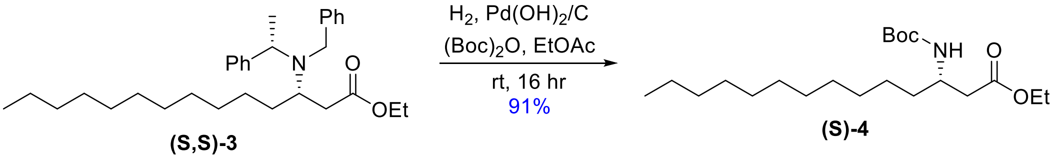

Under a hydrogen atmosphere (balloon), a solution of (*S*)-3-(benzyl((*S*)-1-phenylethyl)amino) tetradecanoate **(*S*,*S*)-3** (1.550 g, 3.33 mmol, 1.00 equiv.), palladium hydroxide (468 mg, 20% w/w on carbon, 0.67 mmol, 20 mol%) and di-*tert*-butyl decarbonate (872 mg, 3.99 mmol, 1.20 equiv.) in ethyl acetate (30 mL) was stirred at room temperature for sixteen hours. The reaction mixture was filtered through celite and the filtrate was concentrated *in vacuo*. The crude product was purified by flash chromatography (19:1 to 9:1 cyclohexane:ethyl acetate) to afford ethyl (*S*)-3-((*tert*-butoxycarbonyl)amino)tetradecanoate (**(*S*)-4**) as a colourless liquid (1.134 g, 3.04 mmol, 91%); **[α]$_D$^{20}** = -12.0 (*c* = 1.00, $CHCl_3$); All other characterisation matched that of **(*R*)-4**.

(*S*)-3-((*tert*-Butoxycarbonyl)amino)tetradecanoic acid ((*S*)-5)):

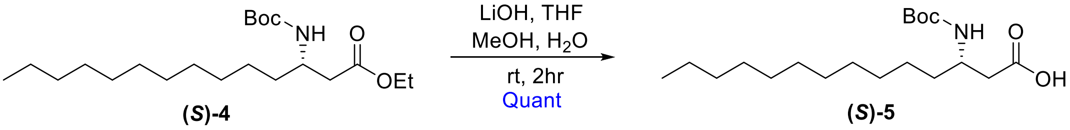

To a solution of ethyl (*S*)-3-((*tert*-butoxycarbonyl)amino)tetradecanoate **(*S*)-4** (1.050 g, 2.83 mmol, 1.00 equiv.) in tetrahydrofuran (26 mL) and methanol (8.5 mL) was added a solution of lithium hydroxide monohydrate (356 mg, 8.48 mmol, 3 equiv.) in water (8.5 mL). The resulting reaction mixture was stirred at room temperature for three hours before being diluted with ethyl acetate (35 mL) and washed with hydrochloric acid solution (1M, 25 mL). The aqueous layer was back extracted with ethyl acetate (2 x 35 mL) and the combined organic layers were washed with water (35 mL) and brine (40 mL), dried over magnesium sulfate, filtered and concentrated *in vacuo* to afford (*S*)-3-((*tert*-butoxycarbonyl)amino)tetradecanoic acid (**(*S*)-5)**) as a white solid (970 mg, 2.83 mmol, Quant.); **MP** = 61-64 ˚C; **[α]$_D$^{20}** = -4.8 (*c* = 0.42, MeOH); All other characterisation matched that of **(*R*)-5**. The enantiopurity of **(*S*)-5** was determined by converting it to the corresponding benzyl ester **(*S*)-6** and then subjecting it to chiral-HPLC analysis.

Benzyl (*S*)-3-((*tert*-butoxycarbonyl)amino)tetradecanoate ((*S*)-6):

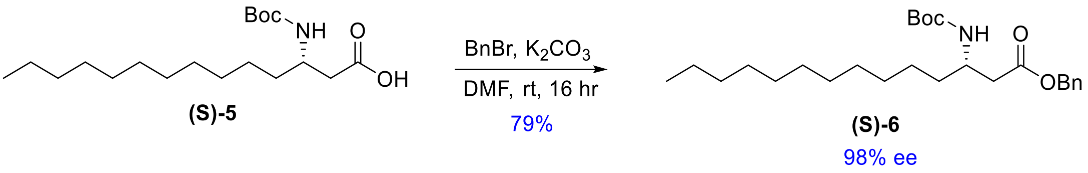

To a solution of (*S*)-3-((*tert*-butoxycarbonyl)amino)tetradecanoic acid **(*S*)-5** (70 mg, 0.20 mmol, 1.00 equiv.) in dimethylformamide (2 mL) was added potassium carbonate (56.3 mg, 0.41 mmol, 2.00 equiv.). Benzyl bromide (29 µL, 41.8 mg, 0.24 mmol, 1.20 equiv.) was then added and the resulting reaction mixture was stirred at room temperature for sixteen hours. The reaction mixture was diluted with water (10 mL) and the product was extracted with dichloromethane (3 x 10 mL). The combined organic layers were washed with brine (15 mL), dried over magnesium sulfate, filtered and concentrated *in vacuo*. The crude product was purified by flash chromatography (6:1 cyclohexane:ethyl acetate) to afford benzyl (*S*)-3-((*tert*-butoxycarbonyl) amino)tetradecanoate (**(*S*)-6**) as a white solid (70.0 mg, 0.16 mmol, 79%); **R$_f$** = 0.4 (6:1 cyclohexane: ethyl acetate).

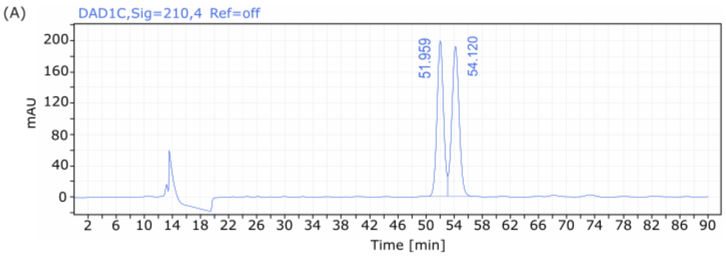

Signal: DAD1C,Sig=210,4 Ref=off

RT [min]	Type	Width [min]	Area	Height	Area%
51.959	BV	2.58	12709.18	198.58	47.83
54.120	VB	3.47	13865.12	191.52	52.17
		**Sum**	**26574.29**		

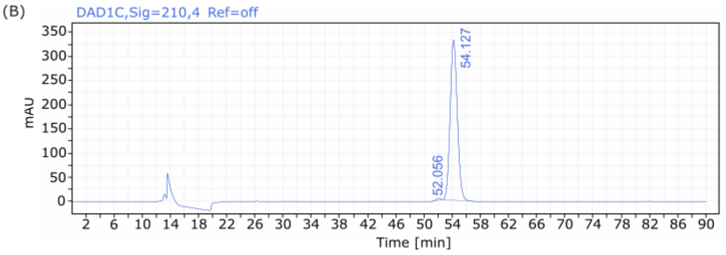

Signal: DAD1C,Sig=210,4 Ref=off

RT [min]	Type	Width [min]	Area	Height	Area%
52.056	BB	1.57	189.87	4.16	0.80
54.127	BB	4.31	23415.08	331.08	99.20
		**Sum**	**23604.96**		

Figure S2: Chiral HPLC analysis of (A) the 1:1 mixture of **(*R*)-6** and **(*S*)-6** and (B) purified **(S)-6**, t_1 = 52.056 min (minor), t_2 = 54.127 min (major). Daicel Chiralpak IA column, 98:2 hexane: isopropanol, 0.25 mL/min, 210 nm.

(*S*)-3-((((9*H*-fluoren-9-yl)methoxy)carbonyl)amino)tetradecanoic acid ((*S*)-7):

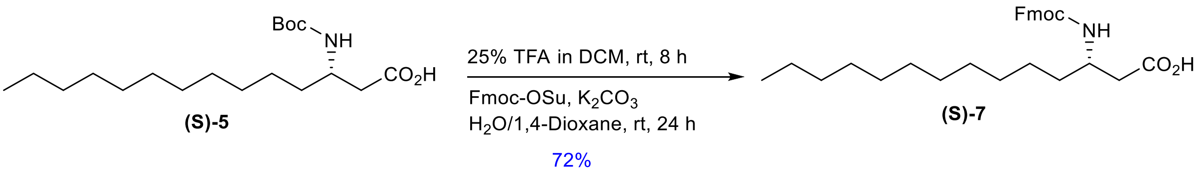

As detailed above for the synthesis of **(*R*)-7**, (*S*)-3-((*tert*-butoxycarbonyl)amino)tetradecanoic acid (**(*S*)-5**, 500 mg, 1.46 mmol, 1.00 equiv.) was dissolved in dichloromethane containing 25% v/v trifluoroacetic acid and the mixture was stirred at room temperature for 8 hours. The solvent

was then removed under reduced pressure and the resulting white solid was dissolved in 10 mL of H_2O:1,4-dioxane (1:1). Potassium carbonate (605 mg, 4,38 mmol, 3.00 equiv.) was added and the mixture was cooled to 0 °C. A solution of Fmoc-OSu (739 mg, 2.19 mmol, 1.50 equiv.) in 1,4-dioxane (5 mL) was added dropwise and the mixture was stirred for 24 hours. The solvent was removed under reduced pressure and 20 mL were added. The solution was acidified with a 5 M solution of hydrochloric acid and extracted with ethyl acetate (3 x 30 mL). The combined organic layers were washed with brine (30 mL) and dried over anhydrous sodium sulfate. On filtration the solvent was removed under reduced pressure and the crude product was purified by flash chromatography (9:1 dichloromethane: methanol) to afford the desired product as a white solid (489 mg, 1.051 mmol, 72%). All characterisation data matched that of **(*R*)-7**.

Solid-phase Peptide Synthesis and cyclisation of (*R*)-8, (*S*)-8, and (*R*)-9:

Method A – SPPS with the cyclisation in liquid-phase:

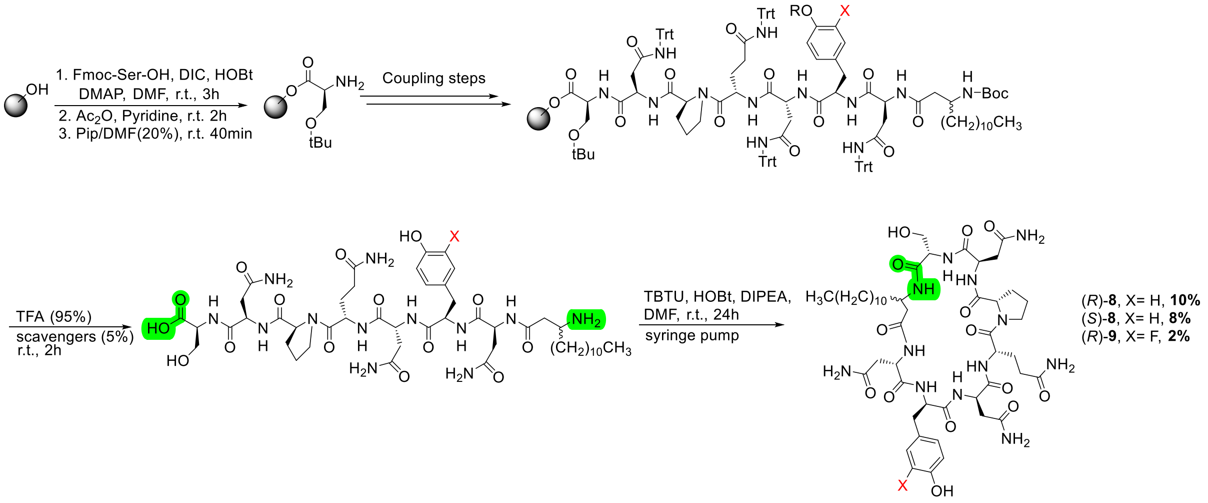

(*R*)-8: Wang resin (100 mg, 0.11 mmol, 1.1 mmol/g, 100–200 mesh) was swollen in DCM (2 mL) for 30 min. Fmoc-Ser(tBu)-OH (211 mg, 0.55 mmol, 5 equiv.), *N,N'*-diisopropylcarbodiimide (DIC, 103 μL, 0.66 mmol, 6 equiv.), hydroxybenzotriazole hydrate (HOBt) (100 mg, 0.66 mmol, 6 equiv.), and 4-dimethylaminopyridine (DMAP) (2 mg, 0.016 mmol, 0.15 equiv.) were dissolved in *N,N*-dimethylformamide (DMF) (2 mL) and added to the resin. The heterogeneous mixture was shaken at 200 rpm (VXR Basic Vibrax shaker) for 6 h at room temperature. After the removal of the solvent via filtration, the resin was washed three times with DMF and DCM (2 mL). The resin was subsequently capped with the addition of a 1:1 solution of acetic anhydride and pyridine (2 mL) with shaking for 2 h. Finally, the resin was washed with DMF and DCM and the Fmoc protecting group was removed with the addition of 20% piperidine in DMF (2 mL) with shaking for 40 min. The deprotection step was repeated and the resin was washed three times with DMF and DCM (2 mL) and dried with a stream of N_2 gas. The coupling and deprotection steps were repeated for the following amino acids: Fmoc-D-Asn(Trt)-OH (197 mg, 0.33 mmol, 3 equiv.), Fmoc-Pro-OH (117 mg, 0.33 mmol, 3 equiv.), Fmoc-Gln(Trt)-OH (202 mg, 0.33 mmol, 3 equiv.), Fmoc-D-Asn(Trt)-OH (197 mg, 0.33 mmol, 3 equiv.), Fmoc-D-Tyr(tBu)-OH (152 mg, 0.33 mmol, 3 equiv.), Fmoc-

L-Asn(Trt)-OH (197 mg, 0.33 mmol, 3 equiv.), ***(R)*-5** (114 mg, 0.33 mmol, 3 equiv.) in the presence of DIC (86 µL, 0.55 mmol, 5 equiv.), HOBt (84 mg, 0.55 mmol, 5 equiv.) and DMAP (2 mg, 0.016 mmol, 0.15 eq.). Coupling and deprotection were routinely monitored via the ninhydrin (Kaiser) test.[4] Cleavage of the resin with a 95% TFA and 5% scavengers (TIPS, H_2O, phenol) solution, followed by concentration under N_2 gas, ether precipitation and centrifugation afforded the crude linear peptide mixture.

TBTU (70 mg ,0.22 mmol, 2 equiv.), HOBt (34 mg, 0.22 mmol, 2 equiv.) and DIPEA (96 µL, 0.55 mmol, 5 equiv.) were dissolved in DMF (30 mL). The crude linear peptide mixture was dissolved in DMF (10 mL) and was added via a syringe pump (Fisherbrand KSD100 Legacy), at a rate of 0.5 mL per hour and the reaction was left at room temperature overnight. The solvent was removed via a flow of N_2 gas and the resulting lipopeptide was purified via HPLC. Lyophilisation of the purified fractions afforded lipopeptide ***(R)*-8** (11 mg, yield 9.6%, purity 97%) as a white solid. Retention time: 16.3 min; **HRMS** (ESI-TOF) calculated for $C_{48}H_{75}N_{12}O_{14}^{+}$ $[M+H]^{+}$: 1043.5520, found: 1043.5518

***(S)*-8:** The synthesis was performed as described above for ***(R)*-8**, with the substitution of the ***(R)*-5** β-amino acid with ***(S)*-5** (114 mg, 0.33 mmol, 3 equiv.). HPLC purification yielded the lipopeptide (9 mg, 7.8% yield, 98% purity) as a white solid. Retention time: 16.0 min; **HRMS** (ESI-TOF) calculated for $C_{48}H_{75}N_{12}O_{14}^{+}$ $[M+H]^{+}$: 1043.5520, found: 1043.5520.

***(R)*-9:** The synthesis was performed as described above for ***(R)*-8**, with the substitution of the Fmoc-D-Tyr(tBu)-OH amino acid with Fmoc-F-Tyr-OH (140 mg, 0.33 mmol, 3 equiv.). HPLC purification yielded the lipopeptide (2 mg, 1.7% yield, 98% purity) as a white solid. Retention time: 16.5 min; **HRMS** (ESI-TOF) calculated for $C_{48}H_{74}FN_{12}O_{14}^{+}$ $[M+H]^{+}$: 1061.5426, found: 1061.5424.

Side-product: Retention time: 15.8 min; **HRMS** (ESI-TOF) calculated for $C_{53}H_{83}FN_{14}O_{14}^{+}$ $[M+H]^{+}$: 1159.6270, found: 1159.6270.

Method B – SPPS with the cyclisation on resin:

Fmoc-Rink Amide resin (100 mg, 0.064 mmol, 0.64 mmol/g, 100–200 mesh) was swollen in DCM (2 mL) for 30 min and the Fmoc protecting group was removed with the addition of 20% piperidine in DMF (2 mL) with shaking for 40 minutes. The deprotection step was repeated and the resin was washed three times with DMF and DCM (2 mL) and dried with a stream of N_2 gas. Fmoc-Glu-ODmab (218 mg, 0.32 mmol, 5 equiv.), DIC (60 µL, 0.384 mmol, 6 equiv.) and HOBt (59 mg, 0.384 mmol, 6 equiv.) were dissolved in DMF (2 mL) and added to the resin. The heterogeneous mixture was shaken at 200 rpm for 6 h at room temperature. After the removal of the solvent via filtration, the resin was washed three times with DMF and DCM (2 mL) and the Fmoc deprotection step was subsequently performed twice, followed by three washes with DMF and DCM (2 mL). The resin was then dried with a stream of N_2 gas. The coupling and deprotection steps were repeated with the following amino acids: Fmoc-D-Asn(Trt)-OH (115 mg, 0.192 mmol, 3 equiv.), Fmoc-D-Tyr(tBu)-OH (88 mg, 0.192 mmol, 3 equiv.), Fmoc-L-Asn(Trt)-OH (115 mg, 0.192 mmol, 3 equiv.), ***(R)*-7** (90 mg, 0.192 mmol, 3 equiv.), Fmoc-Ser(tBu)-OH (74 mg, 0.192 mmol, 3 equiv.), Fmoc-D-Asn(Trt)-OH (115 mg, 0.192 mmol, 3 equiv.), Fmoc-Pro-OH (65 mg, 0.192 mmol, 3 equiv.) in the presence of DIC

(50 µL, 0.32 mmol, 5 equiv.) and HOBt (48 mg, 0.32 mmol, 5 equiv.). Coupling and deprotection were routinely monitored via the ninhydrin (Kaiser) test.[4]

After the final Fmoc deprotection the resin was treated with a 5% hydrazine solution in DMF (2 mL) for 5 minutes and the process was repeated 5 times. The resin was subsequently washed 5 times with DMF (2 mL). Then, the resin was incubated in 3 mL of NaOH (5 mM) in MeOH/H_2O 1:1 for 1 hour to completely elimate the Dmab group. The resin was then washed 3 times with DMF and DCM (2 mL) and was reacted overnight with a 2 mL solution of DIC (20 µL, 0.128 mmol, 2 equiv.) and HOBt (20 mg, 0.128 mmol, 2 equiv.) at room temperature. The cyclisation reaction was repeated one more time with a solution of TBTU (41 mg, 0.128 mmol, 2 equiv.), HOBt (20 mg, 0.128 mmol, 2 equiv.) and DIPEA (56 µL, 0.32 mmol, 5 equiv.) for 3 hours at room temperature, followed by 3 washes with DMF and DCM (2 mL). Cleavage of the resin with a 95% TFA and 5% scavengers (TIPS, H_2O, phenol) solution (1 mL), followed by concentration under N_2 gas, ether precipitation and centrifugation afforded the crude lipopeptide mixture. HPLC purification and lyophilisation of the purified fractions afforded lipopeptide **(*R*)-8** (11.5 mg, 17.2 % yield, 95% purity) as a white solid. Retention time: 16.3 min; **HRMS** (ESI-TOF) calculated for $C_{48}H_{75}N_{12}O_{14}^+$ $[M+H]^+$: 1043.5520, found: 1043.5521

(*S*)-8: The synthesis was performed as described above for **(*R*)-8**, with the substitution of the **(*R*)-7** β-amino acid with **(*S*)-7** (90 mg, 0.192 mmol, 3 equiv.). HPLC purification yielded the lipopeptide (10 mg, 15% yield, 98% purity) as a white solid. Retention time: 16.0 min; **HRMS** (ESI-TOF) calculated for $C_{48}H_{75}N_{12}O_{14}^+$ $[M+H]^+$: 1043.5520, found: 1043.5520.

(*R*)-9: The synthesis was performed as described above for **(*R*)-8**, with the substitution of the Fmoc-D-Tyr(tBu)-OH amino acid with Fmoc-F-Tyr-OH (140 mg, 0.33 mmol, 3 equiv.). HPLC purification yielded the lipopeptide (10 mg, 15% yield, 98% purity) as a white solid. Retention time: 16.5 min; **^{19}F NMR** (376 MHz, $CDCl_3$): -139.1 ppm; **HRMS** (ESI-TOF) calculated for $C_{48}H_{74}FN_{12}O_{14}^+$ $[M+H]^+$: 1061.5426, found: 1061.5423.

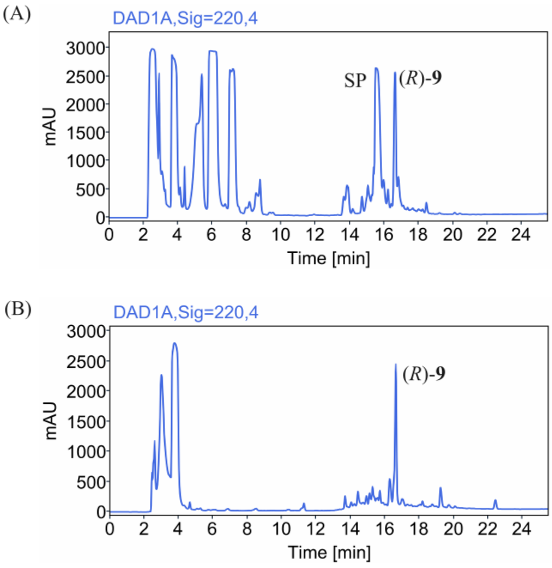

Figure S3: Comparison between the crude HPLC chromatogram of **(*R*)-9** after its synthesis with (A) method A and (B) method B. The side-product (SP) is only present when method A is used. Compounds with a retention time of <10 minutes represent salts and coupling reagents present in the mixtures.

Antifungal susceptibility testing

Assays to determine the Minimum Inhibitory Concentration (MIC) of the lipopeptides were performed against *Candida albicans* and *Fusarium graminearum* in accordance with the CLSI M27-A2[5] and M38-A[6] guidelines respectively. Stock solutions of the lipopeptides were prepared in DMSO and diluted to the desired concentration with RPMI 1640 broth medium. The maximum concentration of DMSO in the 96-well plates was 2.5% (v/v) and control experiments determined that it does not inhibit fungal growth at this concentration.

Briefly, *C. albicans* was grown in Saboraud dextrose agar for 24 hours at 37 °C. Five colonies were picked and were suspended in 5 mL of saline solution (0.85%) and the OD_{600} was adjusted to ~ 0.1, using an Epoch Microplate Spectrophotometer (BioTek). The suspension was vortexed for 15 seconds, diluted 1:100 with saline, followed by a further dilution of 1:20 with RPMI 1640 broth medium (pH=7) and was used as the inoculum.

F. graminearum was grown in Saboraud dextrose agar for 2 days at 35 °C and for 5 days at 28 °C. The fungal culture was then blended with 200 mL of sterile water, 1 mL of the resulting suspension was re-suspended in 5 mL of saline solution (0.85%) and the OD_{600} was adjusted to ~0.15. The suspension was vortexed for 30 seconds, diluted 1:50 with RPMI 1640 broth medium (pH=7) and was used as the inoculum. The assays were performed in sterile flat-bottom polystyrene 96-well plates (Greiner), using the broth microdilution method. For *C. albicans* the 96-well plates were incubated at 35 °C for 24 h with no agitation, while for *F. graminearum* they were incubated for 28 °C for 48 h with no agitation. The well plates were then inspected visually for the presence or absence of fungal growth. The Minimum Inhibitory Concentration (MIC) was defined as the lowest concentration of lipopeptides where complete inhibition of fungal growth was observed. The MIC calculations reflect triplicate experiments. The results for the lipopeptides mixtures in a 1:1 surfactin / iturin A ratio are shown below.

Table S1: MIC calculation for surfactin and for the obtained bioactive lipopeptides in the presence of surfactin (1:1 iturin A / surfactin ratio) against *F. graminearum* and *C. albicans*. The assays were performed in triplicate.

Peptides	MIC (μM)	
	F. graminearum	*C. albicans*
Commercial surfactin	1024	512
(*R*)-**8**: Synthetic iturin A_2	16	64
(*R*)-**8**: Synthetic iturin A_2 / surfactin	32	64
(*R*)-**9**: Monofluorinated iturin A_2 / surfactin	32	64

^{1}H, ^{13}C and ^{19}F NMR Spectra

^{1}H-NMR (400 MHz, $CDCl_3$) ethyl (*E*)-tetradec-2-enoate (**2**)

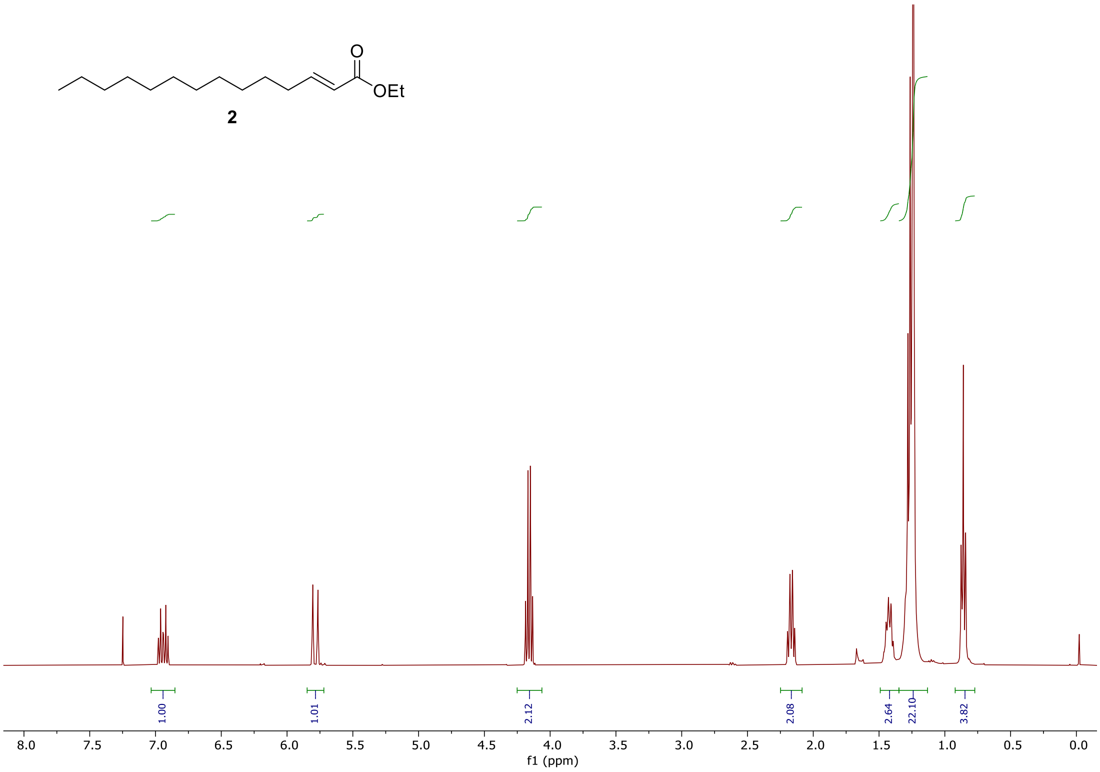

^{1}H-NMR (400 MHz, $CDCl_3$) ethyl (*R*)-3-(benzyl((*R*)-1-phenylethyl)amino)tetradecanoate (**(*R*,*R*)-3)**

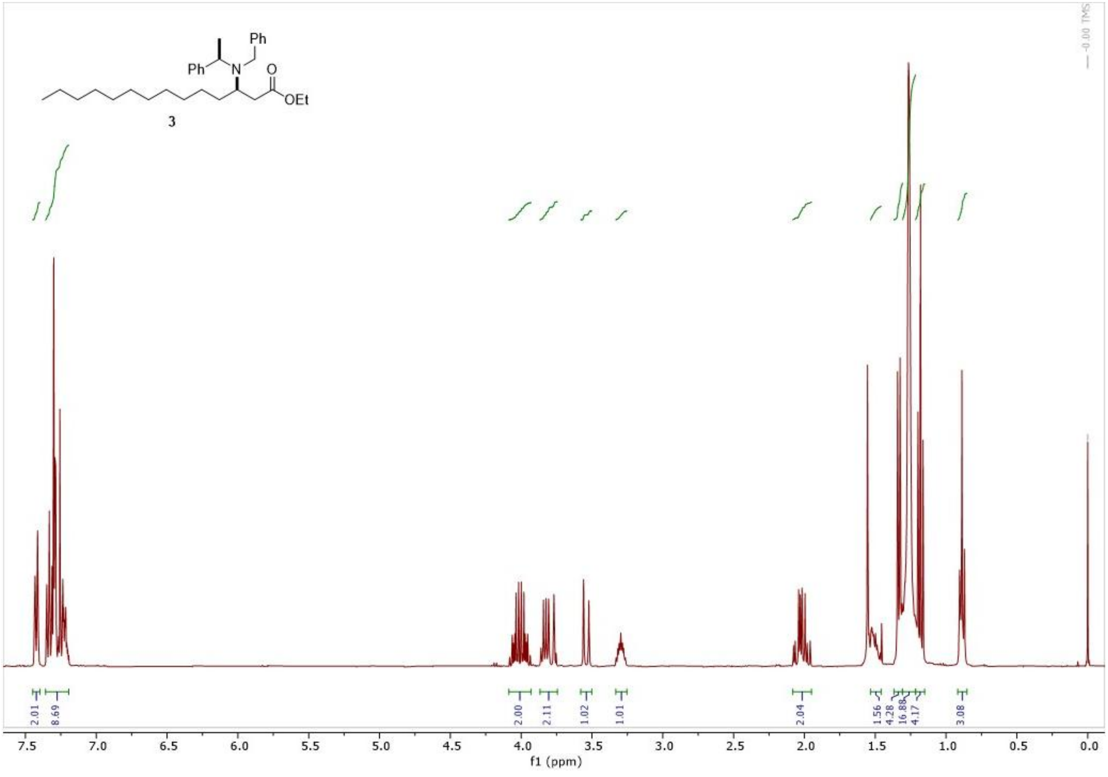

^{13}C-NMR (100 MHz, $CDCl_3$) ethyl (*R*)-3-(benzyl((*R*)-1-phenylethyl)amino)tetradecanoate **((*R*,*R*)-3)**

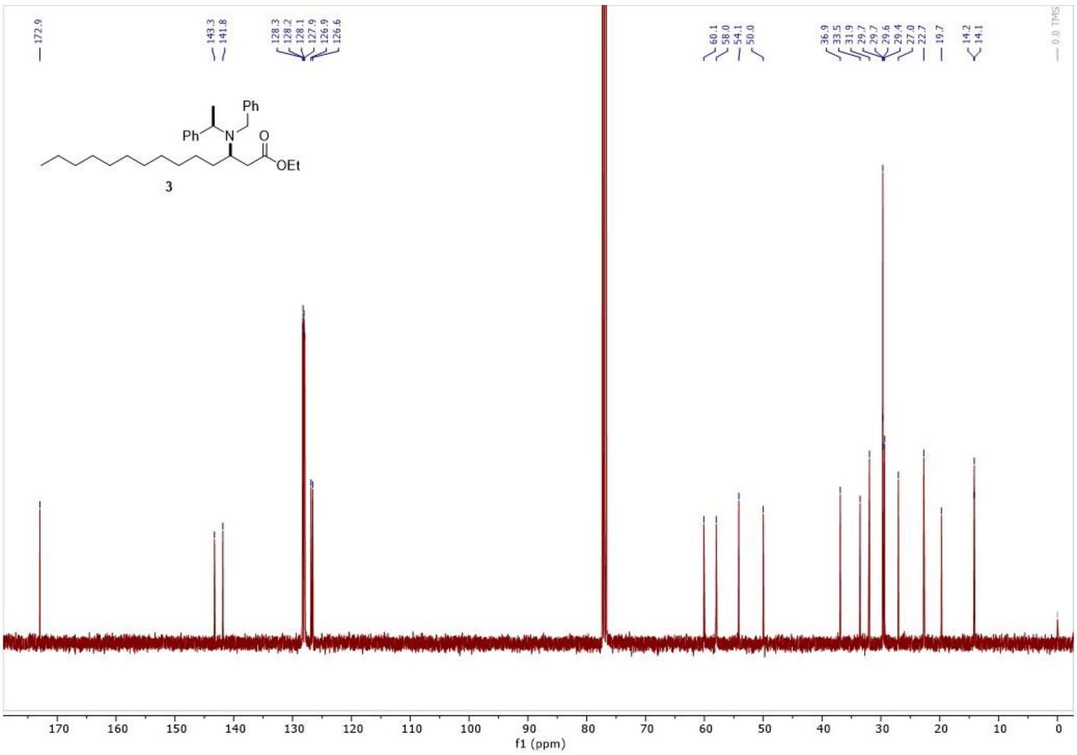

^{1}H-NMR (400 MHz, $CDCl_3$) ethyl (*R*)-3-((*tert*-butoxycarbonyl)amino)tetradecanoate **((*R*)-4)**

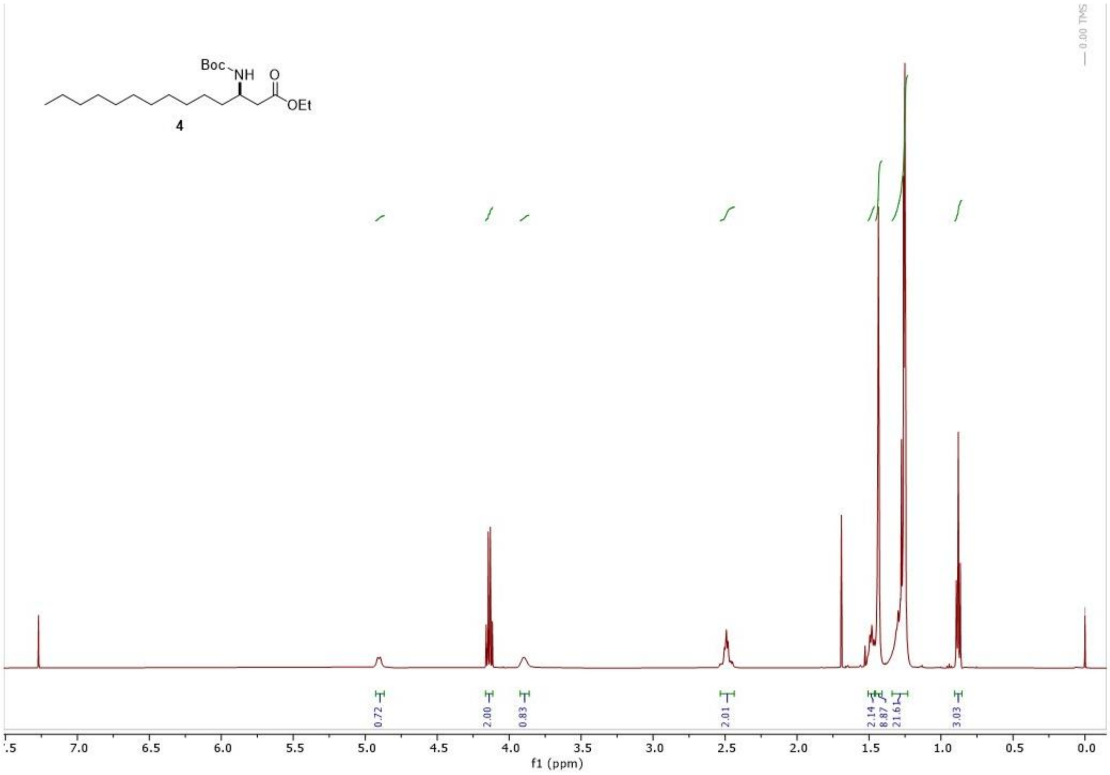

^{13}C-NMR (100 MHz, $CDCl_3$) ethyl (*R*)-3-((*tert*-butoxycarbonyl)amino)tetradecanoate **((*R*)-4)**

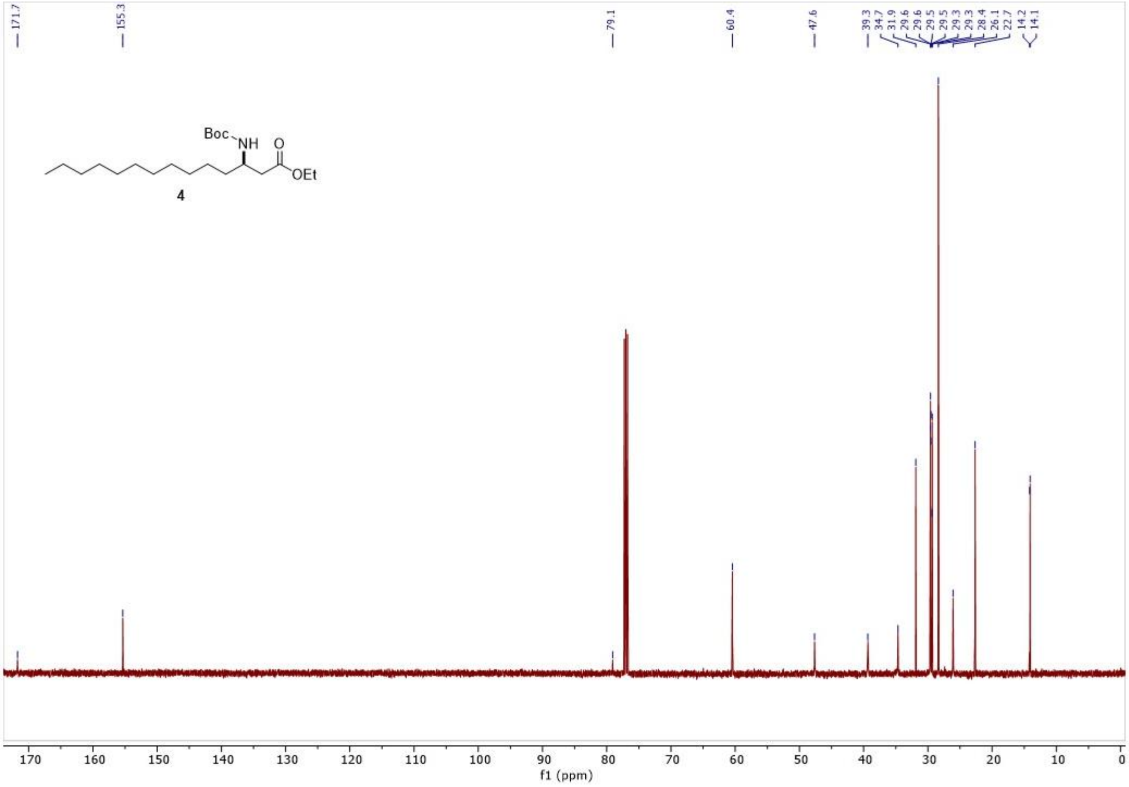

^{1}H-NMR (400 MHz, $CDCl_3$) (*R*)-3-((*tert*-Butoxycarbonyl)amino)tetradecanoic acid **((*R*)-5))**

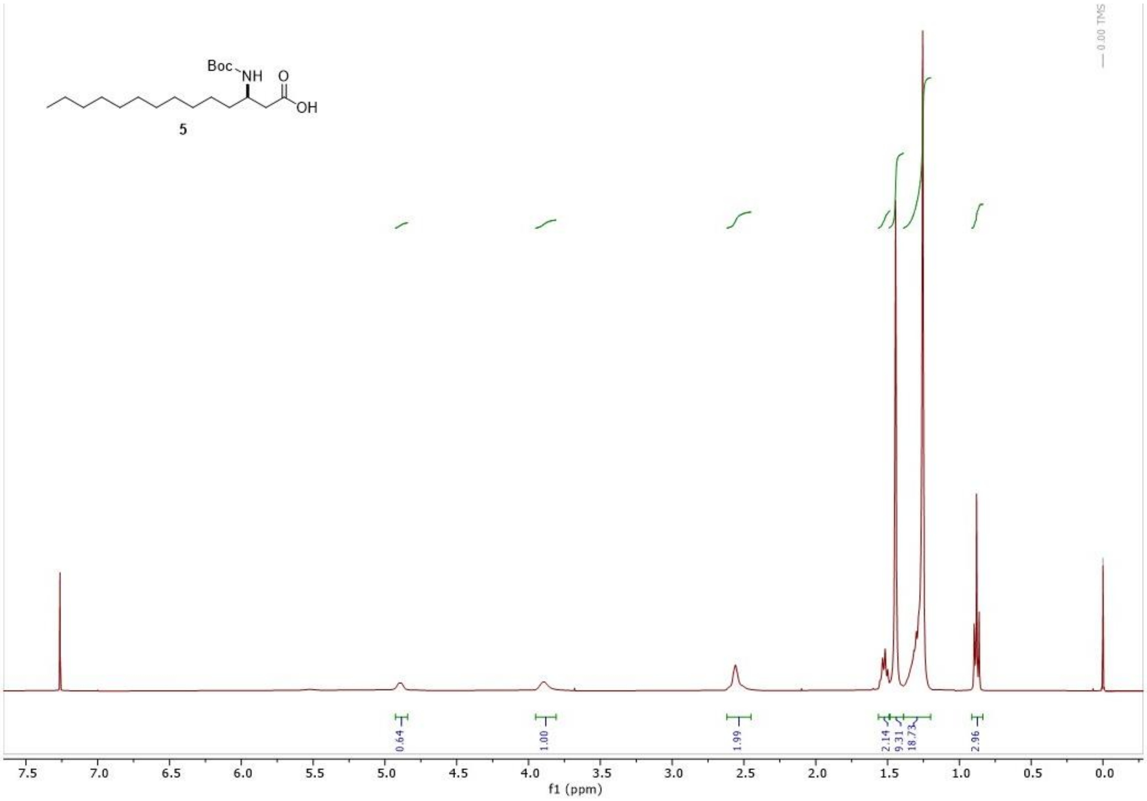

^{13}C-NMR (100 MHz, $CDCl_3$) (*R*)-3-((*tert*-Butoxycarbonyl)amino)tetradecanoic acid **((*R*)-5))**

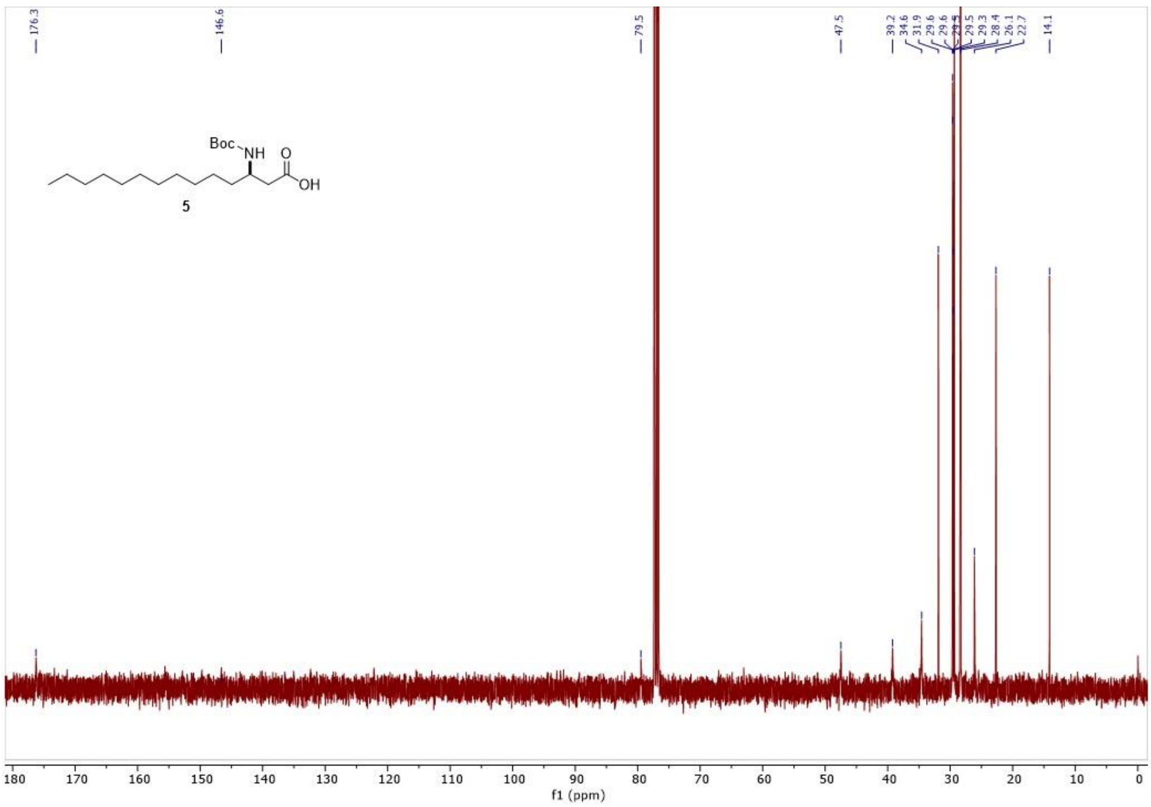

^{1}H-NMR (400 MHz, $CDCl_3$) (*R*)-3-((((9*H*-fluoren-9-yl)methoxy)carbonyl)amino)tetradecanoic acid (**(*R*)-7**)

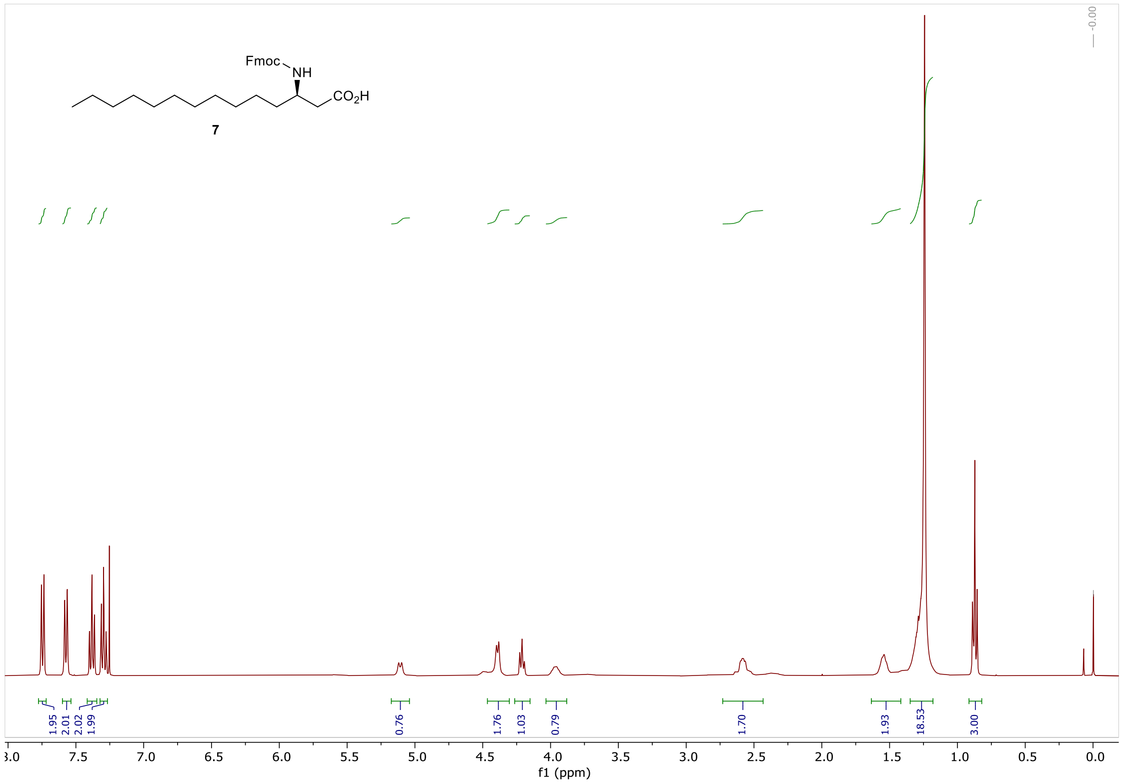

^{13}C-NMR (100 MHz, $CDCl_3$) (*R*)-3-((((9*H*-fluoren-9-yl)methoxy)carbonyl)amino)tetradecanoic acid (**(*R*)-7**):

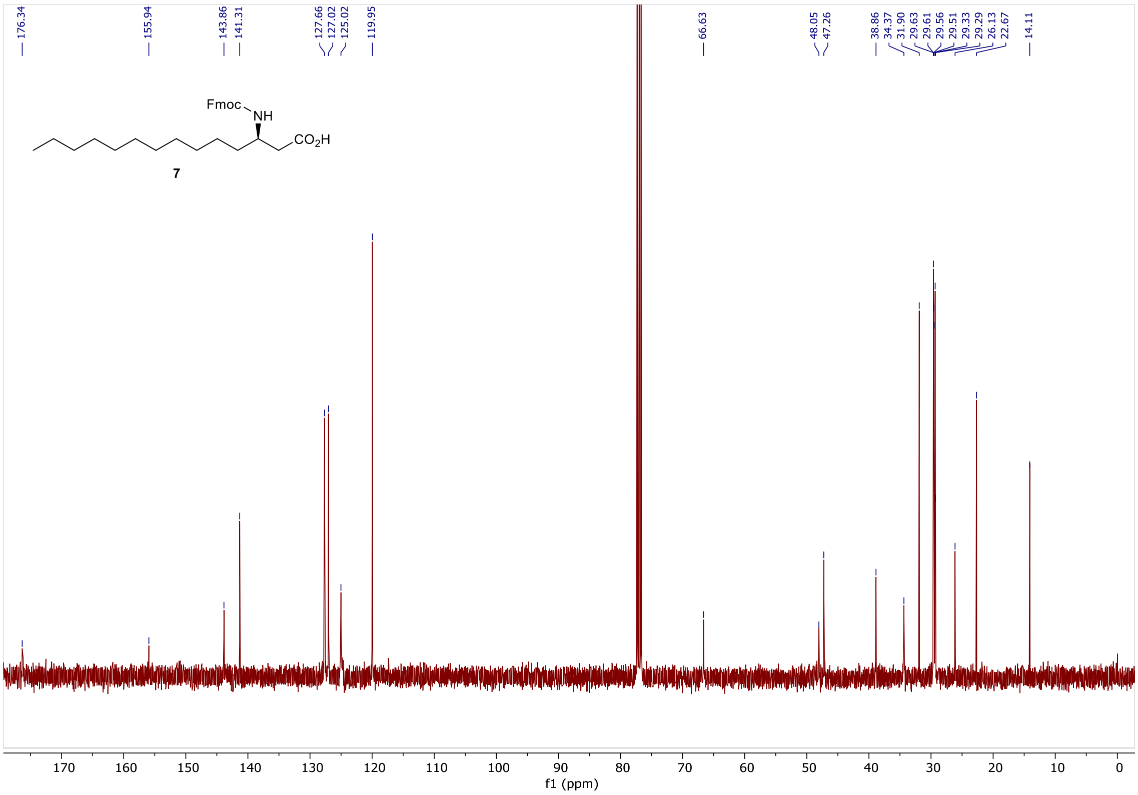

^{19}F-NMR (376 MHz, CD_3OD) Monofluorinated iturin A (**(*R*)-9**)

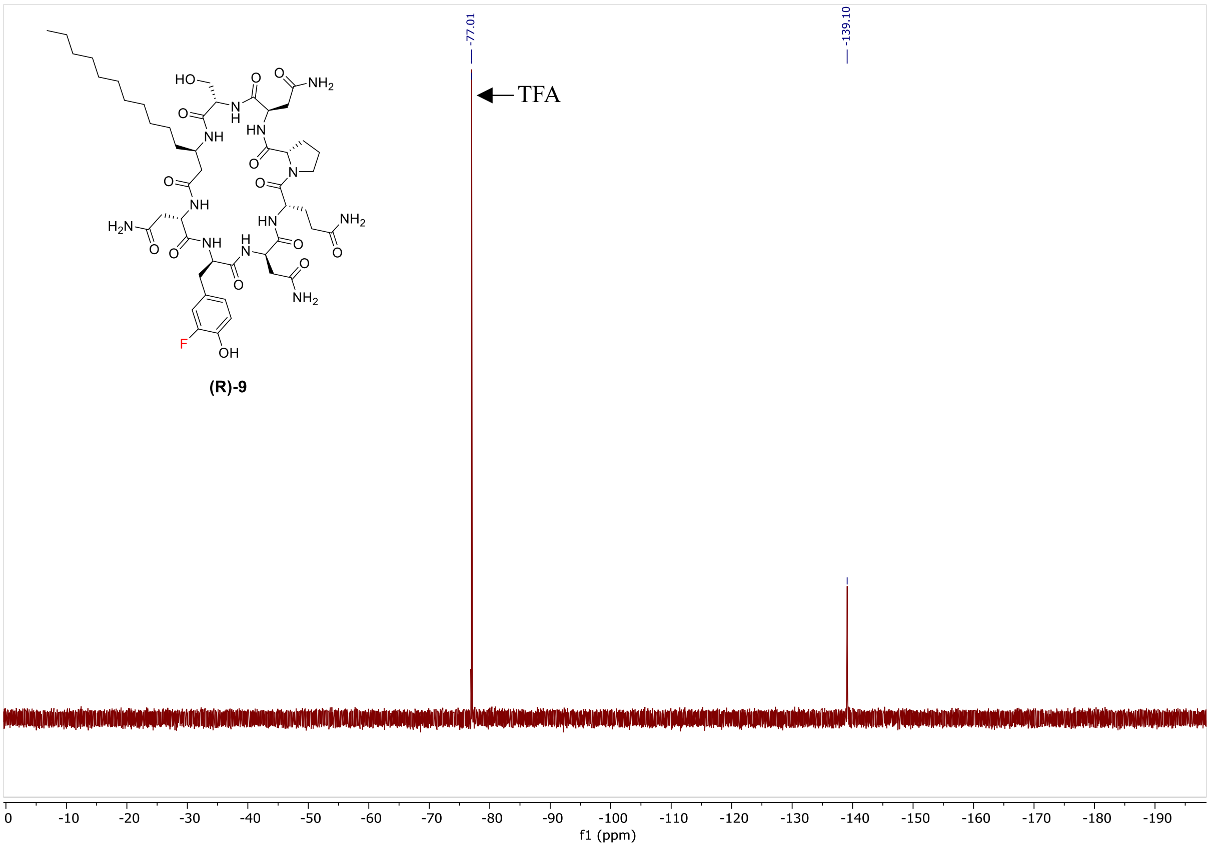

References

[1] L. Corcilius, D. Y. Liu, J. L. Ochoa, *et al.*, *Organic & Biomolecular Chemistry* **2018**, *16*, 5310-5320.
[2] J. Monfray, Y. Gelas-Mialhe, J.-C. Gramain, *et al.*, *Tetrahedron Letters* **2003**, *44*, 5785-5787.
[3] J. M. Bland, *Synthetic Communications* **1995**, *25*, 467-477.
[4] E. Kaiser, R. L. Colescott, C. D. Bossinger, *et al.*, *Analytical Biochemistry* **1970**, *34*, 595-598.
[5] Clinical and Laboratory Standards Institute. Reference Method for Broth Dilution Antifungal Susceptibility Testing of Yeasts; Approved Standard – second edition. CLSI document M27-A2, Wayne, Pa., **2002**.
[6] Clinical and Laboratory Standards Institute. Reference Method for Broth Dilution Antifungal Susceptibility Testing of Filamentous Fungi; Approved Standard - first edition. CLSI document M38-A, Wayne, Pa., **2002**.
